# Assessment Tools, Interventions, and Biopsychosocial Factors Associated With Physical Activity in Older Adults: A Scoping Review

**DOI:** 10.1155/nrp/8838106

**Published:** 2026-06-26

**Authors:** Alberto Gómez-Moreno, Marina García-Gámez, Rocío Badía-Guillén, Rocío Campos-López, MaRosa Iglesias-Parra

**Affiliations:** ^1^ Biomedical Research Institute of Malaga (IBIMA), Malaga, Spain; ^2^ Costa del Sol Primary Care Health District, 29640, Fuengirola, Spain; ^3^ Department of Nursing, Faculty of Health Sciences, University of Málaga, 29017, Málaga, Spain, uma.es

**Keywords:** elderly, frail elderly, older adult, physical activity, questionnaires, reliability, validity

## Abstract

**Introduction:**

The global aging of the population highlights the urgent need to monitor and promote physical activity (PA) among older adults. A multidimensional understanding, encompassing assessment, promotion, and biopsychosocial factors associated with PA, is essential for healthy aging.

**Objectives:**

To map and synthesize the available evidence on PA assessment instruments, intervention strategies, and biopsychosocial factors associated with PA in adults aged 60 years and over.

**Methodology:**

A scoping review was conducted following the JBI methodology and PRISMA‐ScR guidelines. The search included validated and nonvalidated questionnaires, PA promotion interventions, and biopsychosocial factors associated with PA (clinical, psychological, and social domains) in studies published between 2019 and 2026 across community, clinical, and residential settings.

**Results:**

A total of 51 studies were included, showing substantial heterogeneity in sample size, objectives, clinical status, and settings. Twenty different instruments were identified (e.g., PASE, IPAQ, CHAMPS, and VREM), most of them validated. The majority of studies involved populations aged ≥ 65 years and were conducted in the United States, Canada, Australia, and China. Among the biopsychosocial factors associated with PA, frailty (6.45%), cognitive decline (6.45%), and chronic conditions (6.45%) were prominent. Lifestyle‐related factors such as sedentary behavior (11.8%), nutrition (5.37%), and participation in PA programs (16.13%) were also frequent. Outcomes included activities of daily living (9%), quality of life (8.6%), and levels of dependency (6.45%). Intervention‐based studies were less frequently identified compared to observational and methodological studies.

**Conclusion:**

This review highlights the importance of promoting PA to improve functional capacity and quality of life in older adults. Although a wide range of validated tools is available, their application varies depending on context. The limited presence of intervention studies indicates a need for further research focused on structured, multicomponent programs tailored to individual needs within a biopsychosocial framework.

## 1. Introduction

Population aging represents one of the most important challenges; currently, it is estimated that the population over 60 years old will triple in the coming years worldwide. Similarly, in the European Union, it is expected that the population over 80 years old will double during the remaining century [1]. Aging is the result of a physiological process manifested by a progressive deterioration of functional capacity. However, pathological aging entails an unfavorable state for health, which can alter the level of frailty in older adults [2]. It is linked to balance impairment, as it hinders the independent performance of activities of daily living (ADL) and instrumental ADL (IADL), causing an increased risk of falls, which increases the experience of physical frailty, as well as multiplying the level of dependence, reducing the level of quality of life. All of this leads to physical inactivity, considered a risk factor for multiple pathologies that compromise the level of health [3].

The World Health Organization (WHO) defines physical activity (PA) as “any bodily movement produced by skeletal muscles that requires energy expenditure.” For older adults (now defined in this study as ≥ 60 years), the recommendations go beyond mere aerobic accumulation. Current guidelines emphasize the need for multicomponent PA a combination of functional balance and strength training at moderate or greater intensity on 3 or more days a week. These specific activities are crucial for enhancing functional capacity and reducing the risk of falls, distinguishing the requirements of this demographic from those of younger adults [4].

An adequate or “good” level of activity is defined by the attainment of the minimum recommended thresholds that allow older adults to maintain functional independence and an optimal state of physical fitness. According to Rowiński et al. [5], this level is contingent upon engaging in regular PA tailored to individual functional needs, specifically aiming to preserve muscle mass (preventing sarcopenia) and cardiovascular health, which are essential for performing ADL.

Similarly, the WHO states “common physical activities include walking, cycling, biking, playing sports, participating in recreational activities and games; all of which can be done at any level of ability and for the enjoyment of all” [4]. However, in order to maintain a regular level of activity without undesirable events occurring, it is necessary to design activity programs that are tailored to the needs and characteristics of the older population to promote adherence to the program and ensure efficient activity [3].

In order to design certain activity programs correctly, the use of validated instruments is necessary to study their effect on health. The most commonly used tools currently for measuring or estimating the level of activity can be classified into two main groups: estimations through self‐reports or interviews and estimations through PA monitoring [6]. On the one hand, objective measures of PA are based on the use of technologies that measure and record the real‐time of such activity, in addition to estimating the person’s energy expenditure. These devices are responsible for detecting and monitoring movement (heart rate monitors, pedometers, and accelerometers) [7].

Unlike objective PA measurement, questionnaires are often more feasible in epidemiological studies to compare different levels of PA in different countries, as they require lower cost for their use [8]. PA measurement tools are widely used in older adults, as they can be applied in the family environment. Additionally, they are suitable for assessing PA over time and providing valuable information about activities at home and during leisure time [9], and even providing insights into the influence of socioeconomic status on their performance [10].

Early diagnosis of risk factors and providing advice on appropriate PA intervention could reduce healthcare costs related to population aging and promote the health status of the older population. Primary care (PC) is the first point of contact to identify these risk factors with the older adult population. Therefore, the implementation of strategies focused on improving health by promoting adapted PA for the individual would help reduce frailty syndrome in our elders [11].

Therefore, in the healthcare and research fields, validated instruments should be used in terms of measurement properties, reliability, validity, and responsiveness. There are various questionnaires that measure PA. However, PA behaves differently depending on the country and/or region where it is practiced. Therefore, the cultural context and the type of PA in each country should be taken into account when interpreting questions and validating a questionnaire [12].

However, the most recent publications of systematic reviews on the measurement properties of questionnaires in older adults are not very numerous. In 2022, the existing literature on the content and measurement properties of questionnaires and indirect report measuring movement behaviors or their combination in adults and older adults was reviewed [13].

In 2020, the measurement properties of all available self‐administered questionnaires evaluating PA in older adults were compared [9]. At the European level, the measurement properties (reliability, criterion validity, and construct validity) of the most commonly used questionnaires in the general adult population were critically evaluated [12]. Finally, in our country, the psychometric characteristics of PA questionnaires validated in the older 60 years Spanish population were analyzed [3].

The significant demographic shift towards an aging population, accompanied by a rise in life expectancy, has led to an increased prevalence of frailty syndrome among older adults due to factors such as PA [14–16]. Consequently, there is a pressing need to enhance awareness regarding the levels of PA among older individuals. The rationale for utilizing tools to measure PA in the older adult population is well‐founded, as supported by Guthold et al. [8].

In light of these considerations, it is imperative to conduct a scoping review aimed at identifying validated instruments, intervention strategies, and biopsychosocial factors associated with PA that influence PA levels in older adults.

## 2. Material and Methods

**Study design:** A scoping review focusing on studies utilizing PA assessment questionnaires among individuals aged 60 years and above, published in English and/or Spanish within the last 5 years, was conducted.

This scoping review was conducted in accordance with the Preferred Reporting Items for Systematic Reviews and Meta‐Analyses extension for Scoping Reviews (PRISMA‐ScR) guidelines. The review protocol was prospectively registered in the Open Science Framework (OSF) osf.io/5wx8y (https://osf.io/96wbd).

**Search strategy:** Based on the population, concept, context (PCC), methodological framework was used to structure the research question and guide the search strategy.

**Population (P):** Studies including adults aged ≥ 60 years. Studies including participants under this age threshold were excluded.

**Concept (C):** Studies addressing at least one of the following: PA assessment tools, PA promotion interventions, or biopsychosocial factors associated with PA.

**Context (C):** Studies conducted in community, clinical, or residential settings.

**Types of sources of evidence:** This scoping review considered studies of any methodological design in order to comprehensively map the available evidence. Eligible sources included observational studies (cross‐sectional, cohort, and case‐control studies), and experimental and quasiexperimental studies, as well as systematic reviews. Conference abstracts, editorials, and studies lacking sufficient methodological detail were excluded.

**Multiple electronic databases including** PUBMED, SCOPUS, CINHAL, EMBASE, LILACS, ENFISPO, CUIDEN, MEDES, BVS, and PEDro were consulted to identify studies aiming to ascertain the validity and reliability of questionnaires assessing PA performance in the older adult population.

Mesh terms and Boolean operators such as (Questionnaire^∗^ OR survey) AND (“Validity and reliability” OR Assessment) AND (“Physical Activity” OR “Physical Exercise”) AND (Elderl^∗^ OR “Oldest old” OR “Frail elderly”). The search was initially conducted between March and April 2023. Subsequently, an updated search was performed in February 2026 to identify newly published studies. The results of this update were screened and incorporated into the final analysis.

Therefore, the final search period included studies published between 2019 and 2026 (Table 1).

**TABLE 1:** Search strategy in each database.

Database	Search strategy	Search date	Results	Selected
PubMed	(Questionnaire^∗^ OR survey) AND (“Validity and reliability” OR Assessment) AND (“physical Activity” OR “Physical Exercise”) AND (Elderl^∗^ OR “Oldest old” OR “Frail elderly”)	**23/03/2023**	**592**	**130**
PubMed	(Questionnaire^∗^ OR survey) AND (“Validity and reliability” OR Assessment) AND (“physical Activity” OR “Physical Exercise”) AND (Elderl^∗^ OR “Oldest old” OR “Frail elderly”)	**10/02/2026**	**289**	**20**
CINHAL	(Questionnaire^∗^ OR survey) AND (“Validity and reliability” OR Assessment) AND (“physical Activity” OR “Physical Exercise”) AND (Elderl^∗^ OR “Oldest old” OR “Frail elderly”)	**26/03/23**	**148**	**48**
EMBASE	(Questionnaire^∗^ OR survey) AND (“Validity and reliability” OR Assessment) AND (“physical Activity” OR “Physical Exercise”) AND (Elderl^∗^ OR “Oldest old” OR “Frail elderly”)	**26/03/23**	**982**	**110**
EMBASE	(Questionnaire^∗^ OR survey) AND (“Validity and reliability” OR Assessment) AND (“physical Activity” OR “Physical Exercise”) AND (Elderl^∗^ OR “Oldest old” OR “Frail elderly”)	**10/02/2026**	**1056**	**17**
LILACS	(Questionnaire^∗^ OR survey) AND (“Validity and reliability” OR Assessment) AND (“physical Activity” OR “Physical Exercise”) AND (Elderl^∗^ OR “Oldest old” OR “Frail elderly”)	**23/03/23**	**54**	**12**
SCOPUS	(Questionnaire^∗^ OR survey) AND (“Validity and reliability” OR Assessment) AND (“physical Activity” OR “Physical Exercise”) AND (Elderl^∗^ OR “Oldest old” OR “Frail elderly”)	**25/03/23**	**1405**	**210**
ENFISPO	(Questionnaire^∗^ OR survey) AND (“Validity and reliability” OR Assessment) AND (“physical Activity” OR “Physical Exercise”) AND (Elderl^∗^ OR “Oldest old” OR “Frail elderly”)	**26/03/23**	**11**	**3**
BVS	(Questionnaire^∗^ OR survey) AND (“Validity and reliability” OR Assessment) AND (“physical Activity” OR “Physical Exercise”) AND (Elderl^∗^ OR “Oldest old” OR “Frail elderly”)	**26/03/23**	**590**	**77**
PEDro	Questionnaire^∗^ “physical Activity” Elderl^∗^	**27/03/23**	**53**	**3**
MEDES	(“encuestas y cuestionarios”) AND (“validez pruebas”) AND (“ejercicio físico” OR “actividad motora”) AND (ancianos OR “anciano de 80 años y más” OR “anciano frágil”)	**26/03/23**	**1**	**1**
CUIDEN	(“Encuestas“OR”Cuestionarios”) AND (“Evaluación” OR “Validación”) AND (“Actividad física” OR “Ejercicio físico”) AND (“Ancianos”)	**28/03/23**	**4**	**2**
TOTAL DUPLICATES			5181 156	633 **477**

The study’s **inclusion criteria** encompassed articles published in English and Spanish within the last 5 years, focusing on self‐reported and hetero‐administered questionnaires among adult individuals aged 60 years and above. On the contrary, **exclusion criteria** included articles lacking scientific rigor, published over 5 years ago, deviating from the research question, or pertaining to populations under 60 years of age.

**Inclusion criteria** age: Studies were included only if the entire participant sample was aged 60 years or older. This threshold was selected because it represents the clinical transition toward prefrailty and the emergence of health‐related restrictions in PA. Studies reporting a mean age of 60 years but including participants under this age limit were excluded to ensure a specific focus on the biological and social challenges of the older population.

**Exclusion criteria are as follows:**

• Studies where PA is not the primary focus (e.g., studies focused solely on pharmacological outcomes).
• Research focusing on basic science or molecular mechanisms without clinical application.
• Articles with insufficient data regarding the description of assessment tools or intervention protocols.

The concept was structured into three categories. Studies were eligible if they addressed at least one:

1. Assessment tools: Validated or nonvalidated subjective/objective measures.
2. Interventions: Any strategy aimed at PA promotion, ranging from individual clinical prescriptions to community‐based educational campaigns.
3. Factors: Biopsychosocial determinants including clinical conditions (pain and frailty) and social environment factors (social support).

Source selection and screening:

Following the search, all identified citations were uploaded to software Rayyan and duplicates were removed. The screening process was conducted in two stages: (1) title and abstract screening, and (2) full‐text assessment. Both stages were performed by two independent reviewers. Discrepancies were resolved through consensus or by a third reviewer.

Data charting:

Data were extracted from the included studies by two independent reviewers using a charting form developed by the research team. The extracted data included author(s), year of publication, country, study design, population characteristics (age and setting), PA assessment tools, type of interventions, and biopsychosocial factors identified.

Data synthesis:

The synthesis was organized according to the three main concepts: assessment instruments, intervention strategies, and **biopsychosocial factors associated with** PA, within a biopsychosocial framework (clinical, psychological, and social domains).

The extracted data were first organized and presented in tabular format, accompanied by a descriptive narrative to summarize the main characteristics of the included studies.

Subsequently, a qualitative synthesis was conducted by grouping and integrating findings according to the three main concepts of the review: PA assessment tools, intervention strategies, and biopsychosocial factors associated with PA. This approach allowed the identification of patterns, relationships, and key themes across the included studies.

**Data extraction and methodological evaluation**: This scoping review was conducted in accordance with the **JBI methodology** for scoping reviews [17], and the final report was prepared following the **PRISMA-ScR** [18].

Critical reading and quality assessment were performed employing the “quality assessment with diverse studies (QuADS)” tool for observational studies, renowned for its reliability in evaluating multiple studies [19]. Additionally, the “measurement tool to assess systematic reviews (AMSTAR)” tool [20] was employed for systematic review studies.

## 3. Results

**FIGURE 1:**
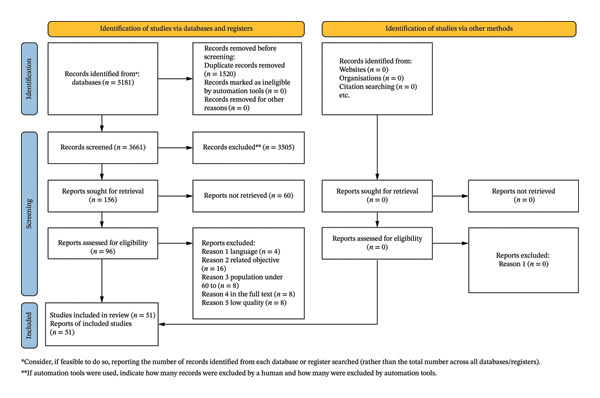
Flowchart.

The search yielded a total of 5181 articles. 1580 duplicate articles were removed. Of the titles and abstracts, 3505 that met the inclusion criteria were eliminated; 156 were reanalyzed, and 60 were removed for not meeting the inclusion criteria after further review. Of these, 96 were selected for critical appraisal. Subsequently, 51 articles were included in the review as depicted in Figure 1.

**FIGURE 2:**
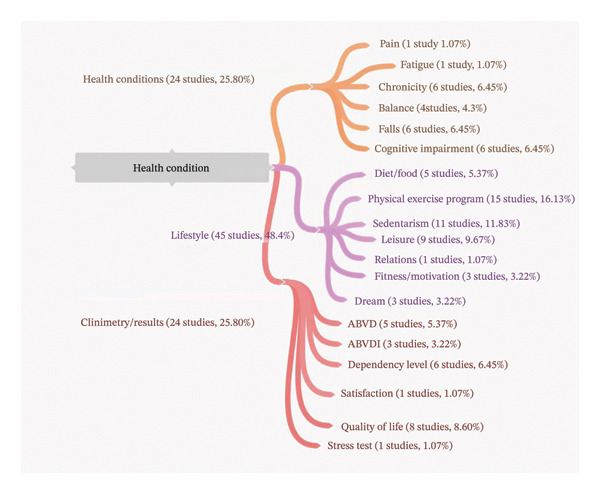
The thematic conceptual structure.

The search strategy yielded a total of 5181 articles, eventually leading to the selection of 51 relevant studies after stringent evaluation. Notably, these studies explored tools for measuring PA in the older adult population while also examining various parameters associated with health status. Among these studies, 24 articles (25.80%) linked PA with specific health conditions, 45 studies (48.4%) delved into lifestyle factors in relation to PA, and 24 studies (25.80%) focused on health outcomes and clinimetry, sometimes examining multiple categories concurrently (see Figure 2).

Regarding health conditions, six studies (6.45%) examined the relationship between PA and cognitive impairment, as evidenced by Alves Silva et al. [21], Gu et al. [22], Öhlin et al. [23], and Van der Wardt [24]. Similarly, frailty in the older adult population was studied in 6 distinct works: Hirase et al. [25], Li et al. [26], Martins et al. [16], Yamada et al. [27], Yamada et al. [28], and da Silva et al. [15].

Furthermore, concerning chronicity, 6 studies (6.45%) explored the association between PA level [29] and frailty level [25, 26, 30–32]. Interestingly, a study linked the risk of falls, frailty level, and pain with PA in older adults [25], while another study highlighted the relationship with the level of fatigue [33]. Moreover, 4 studies (4.30%) identified the level of PA in correlation with the balance state, two of which pertained to the older adult population [14, 34] and the other two focusing on very old individuals [15, 35].

In the realm of lifestyle, 15 studies (16.13%) investigated scales verified through various activities or physical exercise programs in the older adult population. These endeavors were undertaken by Scouvemont et al. [36], Barati et al. [37], Ånfors et al. [38], Brandão et al. [39], Cleland et al. [40], Domingos et al. [41], Król‐Zielińska et al. [42], Król‐Zielińska et al. [43], Pérez et al. [33], Petrella et al. [44], Pigłowska et al. [29], Portegijs et al. [35], Osawa et al. [45], Sattler et al. [9], and Sember et al. [12]. Likewise, 11 articles (11.83%) delved into the consideration of sedentary lifestyle as the main variable, six of which were linked to PA programs for older adults [21, 24, 29, 35, 39, 44, 46, 47] and the remaining focusing on the very older adult population over 80 years of age [45]. On another note, 9 studies (9.67%) were dedicated to investigating the relationship between PA and recreational or leisure activities, involving Alves Faria et al. [1], Gallè et al. [48], Gallè et al. [49], Gu et al. [22], Hatami et al. [50], Osawa et al. [45], and Park and Park [51]. However, a recent study scrutinized the interplay of leisure and sedentary lifestyle [52]. Similarly, 5 studies (5.37%) were dedicated to exploring diet and feeding habits, two of which analyzed the association between PA and nutrition [53, 54]. Another article discussed how recreational activities were linked to dietary choices and social relationships in the older adult population [1]. Additionally, a study uncovered the relationships between PA, nutrition, physical exercise programs, and sedentary lifestyle in older adults [29] along with [55].

Three studies (3.22%) were centered around the relationship of PA with the habitual sleep pattern, with one of them also examining the dietary habits of older adults [53]. Finally, 3 studies (3.22%) delved into the association between motivation, aptitude, and the performance of PA among the older adult [56–58].

Taking a health results‐oriented approach, 24 studies (25.80%) in the clinimetry/results section were scrutinized, with 6 articles (6.45%) exploring the relationship between PA and levels of dependency, ADL, and IADL [10, 21, 34, 39, 59].

Moreover, 8 studies (8.60%) evaluated the association between PA and health‐related quality of life (HRQoL) undertaken by Akosile et al. [14], Brandão et al. [39], Choi et al. [60], Li et al. [26], and Pigłowska et al. [29], with one of these studies also covering the alignment of PA, HRQoL, and the level of satisfaction in older adults [42], as well as Benton and Hutchins [61]. Lastly, a single study dissected the PA levels of the older adult through a stress test conducted by the cardiology service [44].

### 3.1. PA Assessment Instruments

A wide range of instruments have been identified for the assessment of PA in older adults, each varying in psychometric properties, target population, and scope of use. Among the reviewed tools, the **Physical Activity Scale for the Elderly (PASE)** emerged as one of the most widely adopted. Its extensive use is supported by evidence demonstrating high test–retest reliability (*r* > 0.75) and acceptable concurrent validity when compared to established questionnaires such as the **International Physical Activity Questionnaire (IPAQ)**. Moreover, its applicability in clinical populations with complex health conditions, including frailty and multimorbidity, underscores its utility in both clinical and community‐based settings [14, 34, 38, 48, 50]. Recent evidence further supports its discriminative utility: in older adults, a PASE cut‐off ≤ 67 showed sensitivity of 0.76 and specificity of 0.61 for detecting inactivity, with a prevalence of inactivity of 41.37% [31]. Additionally, transcultural adaptation of the French version demonstrated moderate internal consistency (*α* = 0.571), very good test–retest reliability for household (ICC = 0.712), and occupational activities (ICC = 0.955), but low reliability for leisure activities (ICC = 0.163), resulting in overall moderate reliability (ICC = 0.455), with construct validity confirmed in 66.7% of hypotheses [36].

Regarding lifestyle assessment, the **Individual Lifestyle Profile Scale (IHLP)** demonstrated high internal consistency (*α* > 0.80), supporting its reliability in identifying health‐related behaviors among functionally independent older adults [1]. Similarly, the **Persian version of the Rapid Assessment of Physical Activity (RAPA)** showed excellent test–retest reliability (ICC = 0.94) and significant correlations with LEIPAD scores, confirming convergent validity [37].

In terms of objective performance‐based measures, the **Short Physical Performance Battery (SPPB)** is well established in geriatric clinical practice, exhibiting excellent inter‐rater reliability (ICC > 0.90) and strong predictive validity for falls, disability, and frailty [21]. Similarly, the **Timed Up and Go Test (TUG)** was confirmed as a practical and predictive tool for fall risk, with a threshold of 13 s indicating increased vulnerability [39].

The **International Physical Activity Questionnaire—Long Form (IPAQ-L)** has been extensively applied across diverse geographic and cultural contexts. Encompassing various activity domains—occupational, domestic, leisure, and transport—it demonstrated good reliability (ICC ≈ 0.80) and moderate to strong correlations with accelerometry data [16, 21, 39–41]. Its abbreviated version, the **IPAQ-Short Form**, also showed good acceptance among older adults. Although its validity relative to objective measures was moderate (*r* = 0.4–0.6), it remains a feasible option in settings with limited time or resources [38, 62]. An adapted version for individuals aged ≥ 80 years (**IPAQ-E 80+**) demonstrated improved comprehension and response accuracy [23]. Another discriminative analyses showed that **IPAQ-SF** achieved AUC values of 0.45 (nonfrail), 0.40 (prefrail), and 0.67 (frail), with moderate agreement with the FRAIL scale (*κ* = 0.46–0.48) and optimal cut‐off ≤ 322.5, with no significant differences versus PASE [30].

The **Saltin-Grimby Physical Activity Level Scale (SGPALS)** offered a rapid estimate of overall PA levels, showing acceptable correlations with validated self‐report tools [38]. The **Global Physical Activity Questionnaire (GPAQ)**, developed by the WHO, also demonstrated strong reliability (ICC ≈ 0.80) and was particularly valuable in epidemiological and public health surveillance contexts [60].

In the domain of recreational activity assessment, both the **Minnesota Leisure Time Physical Activity Questionnaire (CAFM)** and its Spanish adaptation, the **VREM**, demonstrated high internal consistency (*α* = 0.85), validating their use for capturing leisure‐time PA in older adults [15]. Similarly, the **Yale Physical Activity Survey (YPAS)**, which assesses frequency, intensity, and duration of various activities, showed adequate validity. However, due to its length and complexity, it often requires assisted administration [41].

The **Godin-Shephard Leisure-Time Exercise Questionnaire (GLTEQ)** was positively associated with self‐reported health, biomedical markers, and quality of life [22], while the **Leisure-Time Physical Activity Questionnaire (LTPA)** effectively distinguished between levels of frailty, offering valuable insights into the intensity and variety of PA performed during free time [10].

The **CHAMPS Physical Activity Questionnaire**, developed for older adults, was valued for its sensitivity to change and has been frequently employed in intervention studies due to its strong content validity [12, 42]. In European contexts, the **Nordic Physical Activity Questionnaire—Brief (NPAQ-brief)** showed high reliability (ICC > 0.80) and strong correlations with accelerometer‐derived measures, supporting its application in large‐scale population surveys [62]. Furthermore, a single‐item categorical PA question demonstrated strong convergent validity, with significant correlations with BMI, waist circumference, body fat, vigorous activity, moderate activity, walking, and quality of life (all *p* < 0.001), as well as known‐groups validity confirmed through regression models [61].

The **Healthy Fitness Measurement Scale Version 1.0 (HFMS V1.0)**, which integrates physical and mental health dimensions, demonstrated excellent internal consistency (*α* = 0.92) and a robust factorial structure [56]. Complementarily, a 29‐item PA barriers scale showed three domains (individual, interpersonal, and organizational), four factors explaining 74.14% of variance, good model fit (RMSEA = 0.062; CFI = 0.834), excellent internal consistency (*α* = 0.863), and content validity index of 0.931 [57]. A physical literacy questionnaire comprising 47 items and six subscales demonstrated moderate correlations with leisure activity (*r* = 0.38), activity maintenance (*r* = 0.44), and perceived competence (*r* = 0.58), with Cronbach’s *α* = 0.88 and test–retest reliability = 0.70 [58]. Culturally adapted tools, such as the **Modified Zutphen Physical Activity Questionnaire** for Japanese populations, also proved valid and reliable for retrospective assessment in longitudinal studies [45]. In addition, longitudinal cohort evidence additionally showed that nonexercise PA and exercise habits were significant predictors of incident frailty over 7 years, with adjusted hazard ratios of 0.55, 0.51, and 0.42 depending on activity combination [32].

The **Get Active Questionnaire (GAQ)**, endorsed by the Canadian Society for Exercise Physiology, served as an effective preparticipation screening tool in physically active older populations [44]. Finally, the **Activities Diversity Questionnaire (ADQ)** demonstrated good ecological validity and test–retest reliability, offering a nuanced understanding of the variety and complexity of activities in which older adults engage [23, 63].

Supporting instrument selection decisions, a systematic review of nine self‐report instruments found that IPAQ‐SF and RAPA showed the strongest methodological rigor and usability, while 7‐Day PAR and PASE had good psychometric support but greater administration burden [59].

### 3.2. PA Interventions

Overall, intervention‐based studies were less frequently identified compared to observational and methodological studies. The included studies present different approaches regarding the interventions or procedures applied.

Some studies implemented structured physical exercise programs, such as the randomized controlled trial in which a progressive home‐based exercise program was applied to sedentary older adults, resulting in improvements in functional mobility and quality of life [39].

Other studies adopted a longitudinal or follow‐up approach, examining changes in PA behavior or its relationship with health variables over time, without applying a direct intervention. This includes studies on transitions in PA levels [21] and cohort studies addressing frailty and mortality [27, 28, 32, 45].

In several studies, the main procedure consisted of assessing PA through questionnaires, which were used to analyze their association with variables such as quality of life, frailty, sedentary behavior, and health status [14, 15, 26, 49, 53, 54, 60].

Additionally, many studies focused on the validation, cultural adaptation, and reliability analysis of measurement instruments, including questionnaires such as IPAQ, PASE, and other tools designed for older adults [1, 36, 37, 42, 47, 50, 51, 57, 58].

Other studies evaluated the validity of these instruments by comparing them with objective measures, such as accelerometry or electronic devices, as well as test–retest reliability [23, 38, 40, 41, 62, 64].

Some studies also incorporated the use of technology for PA monitoring, including wearable devices and mobile applications to collect real‐time data [46].

Furthermore, certain studies examined the ability of questionnaires to classify or identify health conditions, such as frailty or physical inactivity, through cut‐off points or discriminative analyses [30, 31].

Finally, some studies analyzed the relationship between PA and other factors, including sociodemographic, nutritional, or lifestyle variables, without applying direct interventions to participants [10, 16, 22, 55].

**FIGURE 3:**
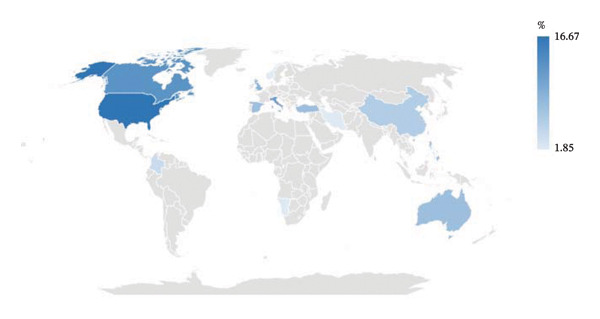
Mapping the distribution of published evidence.

Furthermore, among the analyzed studies, 5 (9.8%) were executed in Japan, while 4 (23.5%) originated from Brazil, China, and Poland. Three studies (17.6%) were conducted in the United Kingdom, Turkey, and EEUU. Additionally, the countries of Portugal, Greece, Iran, Sweden, Italy, and Korea contributed 2 studies each, totaling 23.5%. Furthermore, countries such as Nigeria, Israel, Croatia, Canada, Spain, Finland, Denmark, Belgium, and France each had a single representative study, constituting 17.6% of the analyzed studies. Notably, the remaining 8% of the studies were joint collaborations within the countries of the European Union (Figure 3).

Table 2 presents the distinctive properties of the 51 studies included in this literature review. These have been categorized according to the authors and release date, environment, methodology and purpose, study subjects, tools, and categories used, as well as ratings after critical reading and key findings.

**TABLE 2:** Characteristics of the studies included in the review.

Article	Design	Interventions	Sample	Context	Instruments	Categories	Objectives	Results	Conclusions	Jif quartile
[14]	E. Transversal	No intervention; cross‐sectional comparison of physical activity, fear of falling, and quality of life.	Elderly adult population > 65 years old. *n* = 114 individuals	Nigeria—community and assisted living residence	Physical Activity Scale for the Elderly (PASE). 36‐Item Health Survey Questionnaire (SF‐36).	AF with falls/fear of falling and with quality of life	To compare physical activity (PA), fear of falling, and quality of life among older adults living in nursing homes and communities, and also to determine the correlations between the constructs of each group	Participants in the residences had significantly lower mastery and overall PA scores (*F* = 5.6–103.34; *p* < 0.05) and quality of life (*F* = 11.12–118.05; *p* < 0.05) than community‐dwelling groups. Fear of falling was significantly more prevalent in the assisted living group (*p* < 0.05). There were significant positive correlations (*p* < 0.05) between each pair of PA, fear of falling, and quality of life for both the residential and community housing groups.	Nursing home older adults had lower BP and quality of life scores with a higher prevalence of fear of falling than their community‐dwelling counterparts. There were significant relationships between PA, fear of falling, and quality of life for participants in both groups. Current results may suggest that aging in place ensures better health outcomes than institutionalized aging	Q1 64.1%

[1]	E. Transversal	Questionnaire adaptation and psychometric validation of the Individual Lifestyle Profile Scale.	Elderly adult population > 65 years old. *n* = 300 individuals	Portugal—community/home‐dwelling older adults	“Individual Lifestyle Profile” Scale (IHLP)	Diet and Feeding. Toxic habits. Physical activity and leisure. Relations.	To adapt the Portuguese European context and describe the psychometric properties of the version of the “Individual Lifestyle Profile” (IHLP) scale in a sample of older adults living at home.	After the exploratory factor analysis, a solution was found with four factors that explain a variance of 67.8%. The designation of the factors was changed from the original scale, with the exception of one dimension, and they were called Self‐Management in Health, Social Participation and Group Interaction, Citizenship and Physical Activity. The total internal consistency (Cronbach’s alpha) was 0.858, ranging from 0.666 to 0.860 in the aforementioned factors.	The ILP scale proved to be easy to apply and presented a good index of reliability and validity, based on internal consistency, EFA and AFC. The scale makes it possible to evaluate the lifestyle of older adults, and its use will be aimed at modifying behaviors associated with the negative lifestyles of older adults and their individual needs. 860 in the above factors	Q2 82%

[21]	E. Prospective longitudinal	No intervention; 24‐month follow‐up of physical activity and sedentary behavior transitions.	Elderly adult population > 60 years old. *n* = 374 individuals	Brazil—community‐dwelling older adults	Short Physical Performance Battery (SPPB). International Physical Performance Battery. International Physical Activity Questionnaire (IPAQ ‐ Long).	AF. Level of independence. Instrumental activity.	To verify transition and factors related to the level of physical activity combined with sedentary behavior among community elders followed for 24 months.	Of the 374 elderly people, 61 (16.3%) improved their physical activity and sedentary lifestyle, 226 (60.4%) remained in the same category, and 87 (23.3%) worsened. Unsatisfactory levels of physical activity and sedentary behavior were related to the older age group (*p* = 0.031), absence of professional activity (*p* < 0.001), dependence on instrumental activities of daily living (*p* = 0.013), and poorer physical performance. (*p* < 0.001)	The relationship between sociodemographic and health factors with physical activity and sedentary behavior was demonstrated, reiterating the need for more research on the topic.	Q3 64.1%

[38]	Reliability Study	Test‐retest reliability assessment of physical activity questionnaires in Parkinson’s disease.	Older adult population > 65 years of age. *n* = 49 individuals	Sweden—outpatient/community sample with Parkinson’s disease	International Physical Activity Questionnaire (IPAQ—Short). Physical Activity Scale for Older People (PASE). Saltin–Grimby Physical Activity Level Scale (SGPALS).	AF. Parkinson. Cognitive impairment	To assess the test–retest reliability of PAQs in people with PD without cognitive impairment	Several of the physical activity questionnaires had relatively low test–retest reliability, including the comprehensive questionnaires (IPAQ‐SF and PASE). Total physical activity according to the IPAQ‐SF had a CHF value of 0.46 (95% confidence interval [CI], 0.21–0.66) and the SEM was 2891 MET‐min/week. The total PASE score had an ICC value of 0.66 (95% CI, 0.46–0.79), while the SEM was 30 points. The SGPALS‐last 6 months single item scales (SGPALS‐6 m) and HOET question 1 (HOET‐q1) with longer time frames (6 or 12 months, respectively) showed better results. Weighted kappa values were 0.64 (95% CI, 0.45–0.83) for SGPALS‐6m and 0.60 (95% CI, 0.39–0.80) for HOET‐q1, whereas single‐item questions with a shorter recall period had kappa values < 0.40.	Single‐item questions with a longer time period (6 or 12 months) for physical activity were shown to be more reliable than multi‐item questionnaires such as the IPAQ‐SF and PASE in people with Parkinson’s disease without cognitive impairment.	Q2 77%

[31]	Cross‐sectional	Diagnostic cut‐off analysis to identify physical inactivity using PASE.	Older adult population > 65 years of age. *n* = 394 individuals	Turkey—clinically derived older adult sample/outpatient setting	IPAQ‐SF, PASE	PA. Cut‐off points.	To determine the cut‐off value of the PASE for physical inactivity in older adults.	The physical inactivity cut‐off point for the PASE score in older adults was a score of 67. For identifying physical inactivity, a PASE score of ≤ 67 has a sensitivity of 0.76 and a specificity of 0.61. Among the 394 older adults who participated in the study, 163 were in the inactive group, and 231 were in the active group. The prevalence of physical inactivity was 41.37% in this study.	In the present study, the PASE was found to have moderate sensitivity and specificity in discriminating physical inactivity. It is not a sufficient stand‐alone measure for physical inactivity, so it is recommended that the PASE be included as part of a comprehensive physical inactivity assessment in older adults.	Q1 69.2%

[37]	Methodological	Translation and psychometric validation of the Persian RAPA questionnaire.	Older adult population 60 years. *n* = 300 individuals	Iran—community‐dwelling older adults	RAPA, LEIPAD	PA. Validity. Reliability.	To validate the Persian version of RAPA	Results of comparisons of known groups showed that the mean RAPA score of the older people with greater balance confidence was significantly higher. Significant correlations between most of the scores obtained from both RAPA and the LEIPAD questionnaires confirmed the convergent validity of the questionnaire. Intraclass correlation coefficient (ICC) was as high as 0.94 showing that the test–retest reliability was good.	This study showed that the Persian RAPA is a reliable and valid instrument for measuring physical activity among older individuals in both research and clinical contexts.	Q1 100%

[61]	Cross‐sectional	Validation of a single‐item habitual physical activity question.	Only Women. *n* = 120	United States—community sample	Single‐item PA question; IPAQ‐SF; SF‐36	PA; Self‐report; Validity; HRQoL	To evaluate validity of a single‐item PA question	Mean age was 60 ± 16 years (range 25–89). Most (62%) reported being active or very active. Age was not significantly related to activity levels. Correlation analysis demonstrated good convergent validity. Significant negative correlations were found with body weight, body mass index (BMI), waist circumference, and body fat (all *p* < 0.001). Significant positive correlations were found with vigorous‐intensity activity (*p* < 0.001), moderate‐intensity activity (*p* = 0.004), walking (*p* = 0.005), and quality of life (*p* < 0.001). Good known‐groups validity was demonstrated by significant differences between habitual physical activity levels for body weight, waist circumference, body fat, vigorous activity (all *p* < 0.001), moderate activity (*p* = 0.038), walking (*p* = 0.049), and quality of life (*p* < 0.001). Regression models confirmed known‐groups validity.	A single question with categorical descriptors is valid for a brief clinical assessment of habitual physical activity in women across a wide age range.	Q2 69.2%

[39]	RCT	Progressive semi‐supervised home‐based exercise program.	Elderly adult population > 60 years of age. *n* = 125 individuals	Brazil—community/home‐based intervention	International Physical Activity Questionnaire (IPAQ). Prueba Timed Up and Go (TUG).	AF. Functional mobility. Quality of life. Physical exercise program in the home.	To test the hypothesis that regular practice of a progressive physical exercise program performed at home improves functional mobility and quality of life of sedentary older people in a community	The IG showed an improvement in functional mobility, with a mean reduction in TUG execution time (*p* < 0.01) and an improvement in quality of life (*p* < 0.01) in WHOQOL‐OLD.	Semisupervised physical exercise program at home can be safe and effective in improving functional mobility and quality of life in sedentary older people.	Q2 64.1%

[60]	E. Transversal	No intervention; assessment of sitting time, physical activity, and HRQoL.	Elderly adult population > 65 years of age. *n* = 4276 individuals	Korea—community‐dwelling older adults	International Physical Activity Questionnaire (IPAQ). Global Physical Activity Questionnaire (GPAQ).	PA and sedentary behavior with HRQoL	To examine the association between sitting time and HRQoL by controlling for relevant factors and to assess the combined effect of sitting time and physical activity on HRQoL in older Korean adults	Prolonged sitting time was associated with all dimensions of the EQ‐5D: mobility (odds ratio [OR]: 1.43, 95% confidence interval [CI]: 1.22–1.68), self‐care (OR: 1.65 [95% CI: 1.25–2.17]), usual activities (OR: 2.07 [95% CI]: 1.69–2.52]), pain/discomfort (OR: 1.57 [95% CI: 1.34–1.84]), and anxiety/depression (OR: 1.49 [95% CI: 1.17–1.91]). The long/inactive sitting group had higher ORs for all dimensions of the EQ‐5D than the low sitting/active time group. Prolonged sitting time was associated with low HRQoL in older Korean adults; physical activity may weaken the negative influence of prolonged sitting on HRQoL. The long/inactive sitting group had higher ORs for all dimensions of the EQ‐5D than the low sitting/active time group. Prolonged sitting time was associated with low HRQoL in older Korean adults; physical activity may weaken the negative influence of prolonged sitting on HRQoL. The long/inactive sitting group had higher ORs for all dimensions of the EQ‐5D than the low sitting/active time group. Prolonged sitting time was associated with low HRQoL in older Korean adults; physical activity may weaken the negative influence of prolonged sitting on HRQoL.	Prolonged sitting time and low physical activity are associated with poor overall quality of life in older people	Q2 61.5%

[40]	E. Transversal	Validity study comparing IPAQ with accelerometry for MVPA and sedentary behavior.	Elderly adult population > 60 years, *n* = 253 individuals	United Kingdom—community‐dwelling older adults	International Physical Activity Questionnaire (IPAQ‐long). Accelerometry	PA and sedentary behavior	To assess the validity of the IPAQ (long format) when measuring moderate to vigorous physical activity; To assess the validity of IPAQ (long form) when measuring sedentary behavior in a population of older adults in the United Kingdom (UK) (compared to the Actigraph GT3X).	A sample of 253 older adults (mean age 71.8 years (SD 6.6) and 57% men) was recruited. In total, 226 had valid accelerometer and IPAQ data for MVPA, and 228 had valid data for SB. The results showed that the IPAQ had moderate/acceptable levels of validity (*r* = 0.430–0.557) for MVPA. For SB, there were substantial levels of weekday validity (*r* = 0.702) and reasonable levels of validity (*r* = 0.257) on weekend days. The Bland–Altman analysis showed an inherent measurement error, and most participants tended to underestimate both MVPA and SB.	The results showed that most older adults underestimate their level of MVPA and SB when completing the IPAQ and the above‐average linear relationship shows an error from underestimation to overestimation as the mean increases.	Q2 79.5%

[15]	E. Transversal	No intervention; comparison of frailty, function, and falls by physical activity level.	Elderly adult population > 80 years of age. *n* = 239 individuals	Brazil — community‐dwelling octogenarians	Minnesota Leisure Activities Questionnaire (CAFM/VREM).	PA and Level of Frailty with Prevalence of Falls	To compare differences between frailty, functional capacity, and prevalence of falls among older adults living in the community with respect to their levels of physical activity	The insufficiently active group was the most fragile and had the worst functional performance compared to the other groups. The prevalence of falls was higher in the underactive (60.9%) groups compared to the active (26.4%) and very active (12.7%) groups.	The group of insufficiently active octogenarians showed greater frailty, worse functional capacity, and higher prevalence of falls than the active and very active groups.	Q2 66.6%

[46]	Longitudinal methodological	Sensor‐triggered ecological momentary assessment using Fitbit and smartphone app.	Elderly adult population > 65 years of age. *n* = 88 individuals	Belgium—community‐dwelling older adults	Fitbit; HealthReact app; interviews	PA. Sedentary behavior. EMA.	To examine the patterns in sensor‐triggered EMA protocol adherence (eg, compliance rates), the impact of specific settings (eg, event duration) on the number of prompted surveys, and participants’ experiences with engaging in a sensor‐triggered EMA study.	Participants responded to 81.22% and 79.10% of the EMA surveys in the PA and SB study, respectively. The confirmation rate, defined as the percentage of EMA surveys in which participants confirmed the detected behavior, was 94.16% for PA and 72.40% for SB. Logistic mixed models revealed that with each additional day in the study, the odds of responding to the EMA survey increased significantly by 1.59 times (OR = 1.59, 95% CI: 1.36 to 1.86, *p* < 0.01) in the SB study. This effect was not observed in the PA study. Furthermore, time in the study did not significantly impact the odds of participants confirming to be sedentary (OR = 0.97, 95% CI: 0.92 to 1.02, *p* = 0.28). However, it significantly influenced the odds of confirming PA (OR: 0.81, 95% CI: 0.68 to 0.97, *p* = 0.02), with the likelihood of confirming decreasing by 19% with each additional day in the study. Furthermore, a one‐minute increase in latency (ie, time between last syncing and starting the EMA survey) in the PA study decreased the odds of the participant confirming to be physically active by 20% (OR: 0.80, 95% CI: 0.72 to 0.89, *p* < 0.01). Simulations of the specific EMA settings revealed that reducing the event duration and shorter minimum time intervals between prompts increased the number of EMA surveys. Overall, most participants found smartphone usage to be feasible and rated the HealthReact app as user‐friendly. However, some reported issues, such as not hearing the notification, receiving prompts at an inappropriate time and encountering technical issues. While the majority reported that their behavior remained unchanged due to study participation, some noted an increased awareness of their habits and felt more motivated to engage in PA.	This study demonstrates the potential of sensor‐triggered EMA to capture real‐time data on PA and SB among older adults, showing strong adherence potential with compliance rates of approximately 80%. The SB study had lower confirmation rates than the PA study, due to technical issues and discrepancies between self‐perception and device‐based measurements. Practical recommendations were provided for future studies, including improvements in survey timing, technical reliability, and strategies to reduce latency.	Q1 92.3%

[30]	Transversal	Discriminative performance analysis of IPAQ‐SF and PASE for frailty classification.	Elderly adult population > 65 years of age. *n* = 289 individuals	Turkey—clinically derived cohort/outpatient setting	IPAQ‐SF; PASE; FRAIL Scale	PA; Frailty; Questionnaires	To determine the discriminative performance and optimal cut‐offs of the International Physical Activity Questionnaire‐Short Form (IPAQ‐SF) and the Physical Activity Scale for the Elderly (PASE) for identifying frailty states (nonfrail, prefrail, and frail) in a clinically derived cohort of older adults.	Participants had a mean age of 72.5 ± 7.0 years, and 56.7% were women. Frailty prevalence was 31.1% nonfrail, 29.4% prefrail, and 39.4% frail. For IPAQ‐SF, AUCs were 0.45 (nonfrail), 0.40 (prefrail), and 0.67 (frail); for PASE, AUCs were 0.39 (nonfrail), 0.50 (prefrail), and 0.65 (frail). Macro‐/micro‐AUCs were 0.51/0.53 for IPAQ‐SF and 0.51/0.52 for PASE. No significant differences were found between the two instruments across the frailty categories (DeLong test, *p* > 0.05). At optimal cut‐offs (IPAQ‐SF ≤ 322.5; PASE ≤ 63.6), both questionnaires showed moderate agreement with the FRAIL Scale (*κ* = 0.46–0.48), whereas agreement between the instruments was only low‐to‐moderate (*κ* = 0.32, McNemar *p* = 0.015).	IPAQ‐SF and PASE demonstrated limited ability to discriminate frailty status in older adults, with moderate accuracy in identifying frail individuals and poor discrimination between the nonfrail and prefrail groups. The IPAQ‐SF showed slightly higher specificity, whereas the PASE demonstrated higher sensitivity, indicating that they capture distinct aspects of physical activity behavior but cannot be used interchangeably.	Q1 69.23%

[41]	E. Transversal	Comparison of self‐report questionnaires with accelerometer‐based physical activity estimates.	Elderly population between 64 and 75 years old. *n* = 120 individuals	Portugal—community‐dwelling older adults	International Physical Activity Questionnaire (IPAQ ‐ Long). Yale Physical Activity Survey for Older Adults (YPAS).	AF. Self‐reporting and accelerometry	To compare PA assessed by commonly used self‐report questionnaires versus objective PA assessed by accelerometry, and to describe PA levels and explore their associations with body composition and physical function.	The results highlight a large variation between self‐reported and Xiaomi Mi Band 2 estimates, with poor overall agreement. The biggest difference was found for sedentary time. Low positive correlations were observed for IPAQ (sedentary, vigorous, and total PA) and moderate correlations for vigorous YPAS estimates. Finally, self‐reported, objectively measured PA was differently associated with health outcomes. In summary, although accelerometry has the advantage of being an accurate method, self‐report questionnaires could provide valuable information about the context of the activity.	Accelerometry has the advantage of being an accurate method; self‐report questionnaires could provide valuable information about the context of the activity.	Q2 69.2%

[49]	E. Transversal	No intervention; survey of physical activity determinants in older adults.	Elderly adult population > 65 years of age. *n* = 383 individuals	Italy—community‐dwelling older adults	International Physical Activity Questionnaire (IPAQ).	AF and active lifestyle. Health Promotion Strategies	To assess PA levels among older adults in Bari and assess the association with sociodemographic characteristics, health conditions, and behaviors to identify possible determinants for future PA promotion strategies	The mean time of physical activity was 476.2 ± 297.8 min/week, *p* = 0.08). Weekly sitting time was positively related to age. Attendance at religious or recreational activities was associated with moderate PA.	Educational attainment was positively associated with PA, while owning a dog represented an obstacle to achieving the recommended levels of PA in our population. Participants generally met recommended PA levels, especially men; educational attainment was the main determinant.	Q2 61.5%

[48]	E. Transversal	No intervention; survey of physical activity during the COVID‐19 pandemic.	Elderly adult population > 65 years of age. *n* = 939 individuals	Italy—community‐dwelling older adults	Physical Activity Scale for the Elderly (PASE).	AF y Covid 19 (Pandemica)	To assess the levels and types of PA in a sample of older adults living in the Puglia region during the advanced phases of the COVID‐19 pandemic and to highlight possible relationships between habitual PA behaviors and the sociodemographic characteristics of these individuals.	In total, 68.8% of women surveyed reported a decrease in PA during the pandemic, while 55.1% of men maintained their previous levels (< 0.001). The total PASE score did not differ between gender groups (mean value 91.7 in men vs. 90. *p* = 0.067). However, there were differences in leisure activities, particularly with regard to walking (23.8 ± 14.8 in men vs. 20.2 ± 14.6 in women; *p* = 0.001). Higher PA levels were associated with younger age (OR 0.253; 95% CI 0.192–0.333; *p* = 0.001).	Given that inactivity can affect the health and well‐being of older people, and considering the impact of COVID‐19 confinement on this habit, health promotion strategies to counteract the negative effects of the pandemic should include interventions aimed at increasing PA	Q2 66.6%

[22]	E. Transversal	No intervention; association study of leisure‐time physical activity and brain MRI markers.	Elderly adult population > 65 years old. *n* = 1443	USA—community‐based multiethnic cohort	Godin‐Shephard Leisure Time Exercise Questionnaire (GLTEQ) Magnetic resonance imaging (MRI)	Dementia/Alzheimer’s‐related PA	To examine the association of measures of brain aging assessed by LTPA and MRI in a multiethnic elderly population.	The 1443 study participants had a mean age (SD) of 77.2 (6.4) years; 921 (63.8%) were women; 27.0%, 34.4%, and 36.3% were non‐Hispanic white, non‐Hispanic African American, and Hispanic, respectively, and 27.3% carried the ɛ4 allele of apolipoprotein E (APOE). Compared with the LTPA of nonactive older adults, those with higher LTPA had higher (in cm3) TBV (β [SE], 13.17 [4.42] cm^3^; *p* = 0.003; *p* for trend = 0.006) and greater cortical thickness (β [SE], 0.016 [0.008] mm; *p* = 0.05; *p*para trend = 0.03). The effect size comparing the highest level of LTPA with the nonactive group was equivalent to approximately 3 to 4 years of aging (*β* for 1 year older, −3.06 and −0.005 for TBV and cortical thickness, respectively). A dose‐response association was found, and even the lowest LTPA level had benefits (e.g., TBV: β [SE], 9.03 [4.26] cm^3^; *p* = 0.03) compared to the nonactive group. Adherence to physical activity guidelines for Americans (TBV: β [SE], 18.82 [5.14] cm^3^; *p* < 0.001) and light‐intensity LTPA (TBV: β [SE], 9.26 [4.29] cm^3^; *p* = 0.03) were also associated with larger brain measurements. The association between LTPA and TBV was moderate by race/ethnicity, sex, and APOE status but generally existed across all subgroups. The results remained similar after excluding participants with mild cognitive impairment.	Goldin’s Leisure Time Questionnaire allows for the analysis of LTPA intensity levels and reflects long‐term habitual physical activity. For the elderly population it is more limited, as there is a low correlation with low to moderate PA.	Q1 76.9%

[50]	E. Mixed Descriptive Methodological	Translation and psychometric validation of the Persian PASE.	Elderly population > 65. *n* = 300 individuals	Iran—community‐dwelling older adults	Physical Activity Scale for the Elderly (PASE).	Activity: free time. Physical activity. Activity at home.	To determine the validity and reliability of the translation of the Persian version of the Physical Activity Scale for the Elderly	Reliability was calculated with a Cronbach’s alpha of 0.94, an ICC of 0.99 and a test–retest correlation coefficient of 0.94. The qualitative and quantitative apparent validity of all questions was considered by expert judgment and an SI greater than 1.5. In addition, the CVR and CVI scores for all questions were higher than 0.6 and 0.79, respectively. Confirmatory factor analysis revealed a good fit for the original three‐factor structure.	According to the results, PASE had an acceptable translation, validity, and reliability in the Persian language. Due to the increase in the population of older people, countries are now trying to improve the health of older people and pay attention to their well‐being, it is necessary to value and evaluate physical activity. PASE is a simple and easy‐to‐use scale to assess the physical activity of older people in a few minutes and self‐report.	Q1 66.6%

[25]	E. Transversal	No intervention; assessment of frailty, chronic pain, ADLs, and physical activity.	Elderly population > 65. *n* = 379 individuals	Japan—community‐dwelling older adults	Kihon Checklist (KCL). Accelerometers	AF. Level of dependency. Level of fragility. Level of pain.	To investigate the relationship between frailty and chronic pain, activities of daily living (ADLs) and physical activity in older adults living in the community.	In total, 134 (35.4%) participants met the criteria for frailty; 60.4% of this group had chronic pain. The frail group scored significantly worse on the sensory, emotional, and cognitive aspects of pain, ADLs, and physical activity than the nonfrail group (*p* < 0.05). Age‐ and sex‐adjusted logistic regression analysis showed that sensory and emotional aspects of pain were associated with frailty.	For frail older adults living in the community, chronic pain can negatively influence the sensory, emotional, and cognitive aspects of pain, leading to decreased ADLs and decreased physical activity. Comprehensive pain assessment focused on the sensory and emotional aspects of pain is important to identify frailty among older adults.	Q2 61.5%

[10]	E. Prospective cohort. 15 years	No intervention; prospective cohort assessing socioeconomic status, LTPA, and frailty.	Elderly adult population > 65 years of age. *n* = 601 individuals	Israel—community‐dwelling older adults	Physical Activity and Leisure Time Questionnaire (LTPA). Fried’s phenotype.	PA and frailty with educational and socioeconomic level.	To assess the role of education and income, as well as neighborhood socioeconomic status, in physical activity and subsequent frailty in older adults.	All measures of socioeconomic status (SES) were strongly and positively associated with LTPA (all *p* < 0.001). Eighty‐two participants (14%) were classified as frail at follow‐up. After adjusting for age and sex, and accounting for attrition bias by inverse probability weight, baseline LTPA (OR = 2.77, 95% CI 1.57–4.90, for inactivity; OR = 1.41, 95% CI 0.75–2.68, for insufficient activity, compared with sufficient activity, *p* trend < 0.001) was inversely associated with the incidence of frailty. The association persisted after further adjustment for SES and comorbidity.	Among older people, multiple measures of SES were positively associated with LTPA, which was a strong predictor of a lower risk of later frailty.	Q1 61.5%

[42]	E. Reliability	Polish adaptation and validation of the PAQE questionnaire.	Older adult population between 65 and 89 years old. *n* = 104 individuals	Poland—community‐dwelling older adults	Physical Activity Questionnaire for the Elderly (PAQE‐PL). Acelerometría	Subjective AF and objective AF (accelerometers).	The aim of the study is to evaluate the reliability (test–retest within 1 week, and after 3, 6 and 9 months) and validity (comparing with two questionnaires and an accelerometer) of the Polish adaptation of the Physical Activity Questionnaire for Older Adults (PAQE‐PL)	All test–retest class correlation coefficients (CCI) were significant (ranging from 0.64 to 0.92). Long‐term stability showed significant CHFs (ranging from 0.38 to 0.87) for all participants. In terms of validity, the correlation coefficients obtained were relatively low but statistically significant for all participants between the PAQE‐PL scores and the energy expenditure (*r* between 0.25 and 0.26) measured by the accelerometer. The PAQE‐PL was correlated with almost all CHAMPS‐PL indices, YPAS‐PL energy expenditure, and total time of physical activity. The results suggest that the adaptation of the PAQE‐PL is an acceptable tool for estimating the level of physical activity among older adults in the Polish population. We recommend cautious and well‐thought‐out use of the PAQE‐PL with an older female population. 25 to 0.26) measured by the accelerometer. The PAQE‐PL was correlated with almost all CHAMPS‐PL indices, YPAS‐PL energy expenditure, and total time of physical activity. The results suggest that the adaptation of the PAQE‐PL is an acceptable tool for estimating the level of physical activity among older adults in the Polish population. We recommend cautious and well‐thought‐out use of the PAQE‐PL with an older female population. 25 to 0.26) measured by the accelerometer. The PAQE‐PL was correlated with almost all CHAMPS‐PL indices, YPAS‐PL energy expenditure, and total time of physical activity.	The results suggest that the adaptation of the PAQE‐PL is an acceptable tool for estimating the level of physical activity among older adults in the Polish population. We recommend cautious and well‐thought‐out use of the PAQE‐PL with an older female population, as it measures home PA and leisure‐time PA. It is effective for the elderly, but with drawbacks to measuring together with accelerometers, since the activity at home is of low intensity.	Q2 69.2%

[42]	Reliability Study d	Construct validity and test–retest reliability assessment of the Polish CHAMPS questionnaire.	Elderly adult population > 65 years old. *n* = 104 individuals	Poland—community‐dwelling older adults	CHAMPS Physical Activity Questionnaire.	AF. Health self‐assessment. Life satisfaction and well‐being	To assess the construct validity of the CHAMPS physical activity questionnaire using accelerometers, to assess the criterion validity of the CHAMPS with body composition analysis and the following self‐reported measures: self‐rated health, life satisfaction, and personal well‐being, and to assess test–retest reliability.	The intraclass correlation coefficients of the one‐week test–retest ranged from 0.79 to 0.85 (*r* = 0.33) and caloric expenditure in physical activities of at least moderate intensity (*r* = 0.37) of the CHAMPS physical activity questionnaire. Moderate‐ and higher‐intensity physical activities on the CHAMPS measure were significantly related to total bone mass, self‐rated health, overall life satisfaction, and personal well‐being (*r* ranged from 0.26 to 0.34).	The findings of the study allow us to conclude that the Polish version of the CHAMPS physical activity questionnaire has acceptable reliability and validity for assessing the physical activity of older adults.	Q3 61.5%

[62]	Randomized controlled trial	Concurrent validity study of electronic PA questionnaires versus fitness tracker step counts.	Older adult population > 70 years. *n* = 67 individuals	Denmark—community‐dwelling older adults	IPAQ‐short. NPAQc‐short. Garmin Vivofit 3 Fitness Trackers	PA and sedentary behavior	To investigate the concurrent validity of the International Questionnaire on Physical Activity‐brief (IPAQ‐SF) and the Nordic Questionnaire on Physical Activity‐brief (NPAQ‐brief) compared to objectively measured daily steps among older adults.	The IPAQ‐SF subscales of moderate physical activity (PA), moderate to vigorous PA (MVPA), and sedentary time showed little or no correlation with daily steps. Vigorous PA, moderate PA, and MVPA of the short subscales of the NPAQ showed little or no correlation. Vigorous PA and gait of the IPAQ‐SF subscales showed a regular correlation. Only the metabolic equivalent of IPAQ‐SF task minutes showed a moderate to good correlation with daily steps. The IPAQ‐SF and NPAQ‐short categorization categories of World Health Organization compliance were significantly different, but the magnitudes were small and the distributions indicated problems with categorization.	Concurrent validity is low, as the scores did not reflect objectively measured daily steps.	Q3 61.5%

[26]	E. Transversal	No intervention; association study of physical activity, frailty, fitness, and HRQoL.	Elderly adult population > 65 years of age. *n* = 150 individuals	China–community‐dwelling older adults	Physical Activity Scale for the Elderly (PASE–Short). Senior Fitness Test. Manual grip force test.	HRQoL associated with FA and Frailty	The objectives of this study were; to analyze the effect of demographic characteristics on HRQoL, to explore the correlation between physical activity and HRQoL, to analyze the effect of frailty on HRQoL, and to investigate potential predictors of HRQoL in community‐dwelling older adults.	Dynamic equilibrium was found to be negatively correlated with HRQoL (*r* = −0.40, *p* < 0.01), indicating that participants who spent more time in dynamic equilibrium had worse HRQoL. A significant correlation was found between HRQoL and physical activity. Age and falls affect HRQoL to varying degrees, regardless of the districts in which participants lived. Chronic diseases and BMI have an uncertain but significant effect on HRQoL. In addition, the results of this study showed worse HRQoL scores for participants with poor lower and upper extremity muscle strength/endurance, poor limb flexibility and flexibility, and poor dynamic balance and agility.	HRQoL was found to be significantly affected by upper extremity dysfunction and the prefragile period	Q1 61.5%

[56]	E. Transversal	Psychometric validation of the Healthy Fitness Measurement Scale (HFMS V1.0).	Elderly adult population > 60 years old. *n* = 777 individuals.	China—community‐dwelling older adults	Healthy Fitness Measurement Scale Version 1.0 (HFMS V1.0)	Physical fitness (physical activity). Mental fitness (motivation). Social competence (social resources, support, and social relationships)	To examine the reliability and validity of the Healthy Fitness Measurement Scale Version 1.0 (HFMS V1.0) specifically in older people in China.	The scale had acceptable reliability (Cronbach’s alpha = 0.920, divided half = 0.946, test–retest = 0.878). Exploratory factor analysis showed a KMO value = 0.927 and uncovered 10 factors with a cumulative contribution rate of 65.71% and all factor loads greater than 0.40. The distribution of items was consistent with the initial expectation of the scale. Confirmatory factor analysis indicated a good fit: CMIN/DF = 2.796, RMSEA = 0.048, IFI = 0.914, TLI = 0.902, CFI = 0.913.	HFMS V1.0 was shown to have acceptable reliability and validity ratings for this sample. Overall, HFMS V1.0 is reliable and efficient for measuring the healthy fitness of seniors. It is recommended to use it among older people in other Chinese cities in the future to ensure uniformity and objectivity. This scale can be carried out to assess the effectiveness of public health measures in improving the level of healthy fitness of older people and optimizing public health policies.	Q1 94.8%

[32]	Prospective cohort	No intervention; 7‐year cohort comparing nonexercise physical activity and exercise habits.	Elderly adult population > 65 years old. *n* = 1288 individuals.	Japan—community‐dwelling older adults	CHS index, GPAQ, self‐report. NEPA	PA. Frailty. Cohort.	To compare the impact of moderate‐to‐vigorous‐intensity NEPA and exercise habit (EH) on frailty among community‐dwelling older adults.	Compared to participants with no NEPA nor EH, those with NEPA only, with EH only, and with both showed significantly lower adjusted odds ratio (95%CI) of frailty: 0.29 (0.16–0.52), 0.21 (0.11–0.41) and 0.21 (0.12–0.36). NEPA and EH at baseline were predictor variables for new‐onset frailty during the 7‐year follow‐up period, with adjusted hazard ratios (95% CI) of 0.55 (0.33–0.92) for NEPA only, 0.51 (0.29–0.90) for EH only, and 0.42 (0.25–0.70) for both. No significant differences were observed between the associations of NEPA and EH with frailty.	NEPA is associated with lower frailty risk in older adults, with a similar but nonadditive effect to that of EH. These findings highlight the importance of NEPA for frailty prevention, particularly for those not engaged in formal exercise programs.	Q1 69.2%

[16]	E. Transverse	Isotemporal substitution analysis replacing sleep or sedentary time with moderate physical activity.	Adult older population > 60 years *n* = 456 individuals	Brazil—community‐dwelling older adults	International Physical Activity Questionnaire (IPAQ ‐ Long). Pittsburgh Sleep Quality Index.	PA. Dream. Level of Fragility.	To examine the hypothetical effect of replacing time spent in sleep or BS with equivalent time spent performing moderate or vigorous PA on frailty syndrome in the older population.	Substitution of 60 min/day of BS (prevalence ratio, PR = 0.52; 95% confidence interval, CI: 0.28–0.96) or sleep (PR = 0.52; 95% CI 0.27–0.98) per 60 min/day of moderate exercise PA (MPA) was associated with a 48% reduction in the prevalence of frailty syndrome.	Replacing sitting or sleeping time with the same amount of MPA time may reduce the risk of frailty syndrome	Q2 61.5%

[59]	Systematic review	Systematic review of initial validation studies of self‐report physical activity instruments.	9 studies	United States—not applicable (systematic review)	IPAQ, GPAQ, PASE, Baecke, YPAS, CHAMPS, BRFSS, Stanford Recall	PA. Measurement tools. Validation.	To examine the methodological rigor and psychometric reporting of self‐report physical activity (SRPA) instruments for adults, focusing on their initial validation studies.	Nine SRPA instruments were identified and evaluated. The International Physical Activity Questionnaire Short Form and Rapid Assessment of Physical Activity demonstrated the highest methodological rigor, with strong validity, ease of use, and broad applicability. The 7‐Day Physical Activity Recall and Physical Activity Scale for the Elderly had good psychometric support but were more burdensome to administer. Common limitations included incomplete reporting of reliability and dimensionality.	The SRPA instruments vary in quality and feasibility. Selection should align with study objectives, population characteristics, and the specific psychometric strengths and limitations identified in each instrument.	Q1 87.5%

[47]	Methodological	Turkish adaptation and psychometric validation of the LASA‐SBQ.	Adult older population > 65 years *n* = 100 individuals	Turkey—community‐dwelling older adults	LASA‐SBQ, SBQ, Epworth, IPAQ‐SF	Sedentary behavior. Validation. Reliability.	To adapt the Longitudinal Aging Study Amsterdam Sedentary Behavior Questionnaire (LASA‐SBQ) into Turkish. Turkish translation, validity and reliability studies were performed.	The mean time spent by the participants as sedentary in a week was 9.390 ± 3.733 h. There was a correlation between the LASA‐SBQ and the total score of the SBQ (Pearson *r* = 0.757; *p* < 0.01). The test–retest reliability of the LASA‐SBQ was examined and the intraclass correlation coefficient was found to be 0.978. In order to examine the validity of the questionnaire together with the SBQ, Bland‐Altman analysis was performed and a graph was drawn. Bland‐Altman analysis shows that the validity of the questionnaire is high.	The LASA‐SBQ was translated into Turkish and culturally adapted. The psychometric properties of the questionnaire were examined and validity and reliability analyses were performed. The Turkish version of the LASA‐SBQ is a valid and reliable scale and is suitable for use in scientific research.	Q1 92.3

[55]	Cross‐sectional	No intervention; assessment of grandparenting effects on physical activity, diet, and quality of life.	Adult older population > 65 years *n* = 152 individuals	Greece—community‐dwelling older adults	IPAQ, MedDiet, WHOQOL‐BREF	Physical activity. Diet. Quality of life.	to assess the effect of grandparenting on physical activity, nutrition, and quality of life of older people.	Significant correlations were found concerning the care of grandchildren with physical activity, eating habits, and quality of life of older adults. The most notable results of regression analysis, regarding grandparenting effect, were the number of grandchildren being taken care of, which had a statistically significant negative effect on dietary habits, physical activity and quality of life, and being the only caregivers, which was positively associated with dietary habits, but negatively with the two other outcomes (all *p* < 0.01).	The results showed that different aspects of informal childcare had statistically significant effects on dietary habits, physical activity, and overall quality of life in older people.	Q2 69.2%

[23]	Cross‐sectional study	Concurrent validity study of IPAQ‐E 80+ versus accelerometry.	Elderly adult population > 80 years of age. *n* = 76 individuals	Sweden—community‐dwelling adults aged 80+	International Physical Activity Questionnaire for People Over 80 (IPAQ‐E 80+). Accelerometry.	PA and dementia	To evaluate the concurrent validity of the IPAQ‐E 80+ in a sample of very elderly people using accelerometer data, with relevant subgroup analyses.	The IPAQ‐E 80+ was correlated with accelerometer measurements of total inactive (*r* = 0.55, *p* < 0.001), sedentary (*r* = 0.28, *p* = 0.015), walkers (*r* = 0.54 *p* < 0.001) and total active. ‐ (*r* = 0.60, *p* < 0.001) times, but not with measures of walking intensity or physical activity; MV walking (*r* = 0.06, *p* = 0.58), MVPA (*r* = 0.17, *p* = 0.13). In this study, the IPAQ‐E 80+ showed medium to substantial correlations with accelerometers and thus appears capable of classifying very elderly people according to PA levels (total inactive time, sedentary and total active time, and walking time).	The IPAQ‐E 80+ shows promise for use in studies investigating associations between activity behavior and health in this population. Given the option of indirect confirmation for respondents with cognitive impairments, the IPAQ‐E 80+ may also be suitable for individuals with dementia (the option to verify the response with family members or care staff is provided)	Q2 82%

[45]	E. Prospective cohort. 6 years	No intervention; prospective cohort examining physical activity, biomarkers, and mortality.	Elderly adult population > 85 years of age. *n* = 441 individuals	Japan—community‐dwelling adults aged 85+	Modified Zutphen Physical Activity Questionnaire.	AF. Biomarkers of mortality.	To examine whether selected biomarkers mediate the association between PA and all‐cause mortality at 6 years in very older people living in the community.	A curvilinear relationship was observed in the association between baseline PA and all‐cause mortality. Compared to the inactive (0 MET∗h/week), a slight amount of PA was associated with a lower risk of mortality. Compared with the highest tertile of PA (11.2 MET∗h/week), higher PA did not reduce the risk of death. Circulating albumin and cholinesterase levels measured the association between baseline physical activity and all‐cause mortality (median ratio, 54%, both; *p* < 0.05).	Compared with total inactivity, mild PA reduces the risk of all‐cause mortality in the very elderly population. Mediating analysis suggests that protein synthesis in the liver may mediate the association between PsA and all‐cause mortality	Q2 77%

[53]	E. Transversal	No intervention; association study of nutritional status, physical activity, sleep, and HRQoL.	Elderly adult population > 65 years of age. *n* = 3405 individuals	Greece—community‐dwelling older adults	International Physical Activity Questionnaire (IPAQ). Pittsburgh Sleep Quality Index (PSQI).	AF. Nutrition. HRQoL. Dream	To explore associations between nutritional status and health‐related quality of life, physical activity, and sleep quality in exclusively Caucasian older adults in Greece who did not have any serious illness	(10.4%) of the participants were classified as malnourished, while 35.6% were “at risk of malnutrition” (*p* = 0.0011) and had better quality of life (*p* = 0.0135), as well as better sleep quality (*p* = 0.0202).	The interrelationships between good nutritional status, high‐quality sleep, an active lifestyle and a good quality of life are highlighted	Q1 61.5%

[51]	E. Mixed	Development and psychometric validation of the Leisure Valuation Assessment Tool for the Elderly.	Elderly adult population > 60 years old. *n* = 454 individuals	Korea—community‐dwelling older adults	Leisure AssessmentTool for the Elderly (LVAT‐E).	AF & Leisure	To develop a “Leisure Assessment Tool for Older People” (LVAT‐E) to revitalize leisure activities for older people living in the community and to verify the suitability, reliability, and validity of this model assessment tool for older people living in the community.	The research method, literature review, and Delphi survey were conducted for the expert panel. Then, the items of value of leisure and participatory leisure activity were derived to form the evaluation items. The two Delphi surveys revealed 38 items assessing the value of leisure and 41 items of participating leisure activities. We tried to verify the suitability and validity of the model of the items that evaluated the value of leisure by means of confirmatory factor analysis. The verification showed a good fit. According to the result of the intensive validity test, the AVE values (mean variance extracted) were 66 for physical leisure activities, 65 for emotional leisure activities and 65 for social leisure activities. Conceptual reliability was 0.96 for physical leisure activities, 0.95 for emotional leisure activities and 0.96 for social leisure activities. Regarding internal consistency for the verification of reliability, Cronbach’s alpha values for physical leisure, emotional leisure and social leisure activities were 0.909, 0.925 and 0.955, respectively. Therefore, the items were highly interrelated and homogeneous tests measuring the same characteristics. The assessment tool can be used to identify useful information about the leisure activities of older people and to activate leisure activities for older people. 0.925 and 0.955, respectively. Therefore, the items were highly interrelated and homogeneous tests measuring the same characteristics.	The assessment tool can be used to identify useful information about the leisure activities of older people and to activate leisure activities for older people. High reliability. Positive correlation. 45 items with 3 categories (Physical Activity, Emotional Leisure, Social Leisure).	Q2 69.2%

[33]	E. Reliability	Validation of the Spanish Pittsburgh Fatigability Scale against physical performance and activity measures.	Elderly adult population > 70 years old. *n* = 79	Spain—community sample of inactive older adults	Pittsburgh Fatigability Scale (PFS)	AF and fatigue	To validate the Spanish version of the PFS by assessing convergent validity against various measures of physical performance, physical activity, physical function, and disability in a sample of inactive older adults.	Higher physical PFS scores were inversely associated with short battery of physical performance (*r* = −0.5, *p* < 0.001) and correlated from weak to moderate with gait speed (*r* = −0.38, *p* = 0.001) and self‐reported weekly walking time (*r* = −0.24, *p* = 0.035).	The PFS is a novel and brief instrument to assess fatigability in Spanish‐speaking older adults, with good convergent validity against measures of physical performance.	Q2 61.4%

[44]	E. pre–post	Psychometric evaluation of the Get Active Questionnaire against treadmill stress testing.	Elderly adult population > 60 years old. *n* = 120 individuals	Canada—community‐dwelling older adults	Cuestionario Get Active (GAQ)	AF and stress test	To assess the psychometric properties (test–retest reliability and criterion‐related validity) of the Get Active Questionnaire (GAQ; Canadian Society of Exercise Physiology (CSEP) 2017) in the community in older adults	In the present sample (with responses from relatively healthy older adults), self‐completion of the GAQ had a low risk assessment sensitivity compared to the treadmill stress test. This was supported by poor positive predictive value and poor false negative probability. However, consistent with the reliability and validity of the items, the GAQ had good specificity in the risk assessment.	The GAQ shows promise in assessing physical activity in older adults to exercise safely. However, the lack of accuracy in detecting physical activity in at‐risk populations requires further evaluation.	Q2 66.6%

[29]	E. Cases and controls	No intervention; case‐control comparison of determinants of quality of life in community and nursing home residents.	Elderly adult population > 60 years of age. *n* = 200 individuals	Poland—community and nursing home	The Seven Day Recall Physical Activity. Questionnaire. Stanford Usual Activity Questionnaire.	AF. Level of Nutrition. Chronic disease. Quality of life.	To examine and compare the relationship between nutritional status, level of physical activity (PA), concomitant chronic diseases, and quality of life (QoL) in older people living in resident communities (CD) and nursing homes (NH).	*p* < 0.05), but there were no differences within the five dimensions of quality of life. In NH patients, the VAS scale was not correlated with any of the variables that assess nutritional status and body composition, while in the CD group, it was positively correlated with MNA (rS = 0.36; *p* < 0.001), % FFM (rS = 0.22; *p* < 0.05), body density (rS = 0.22; *p* < 0.05). and negatively with % FM (rS = −0, 22; *p* < 0.05). In an institutional setting, only comorbidities (mainly urinary incontinence) were found to be independent determinants of quality of life. In the community, the independent determinants of quality of life, in addition to comorbidities (mainly ischemic heart disease), were nutritional status or BP	The determinants of quality of life are different depending on the living environment of older adults. Adequate nutritional status and beneficial PA behaviors are crucial for a higher quality of life for the elderly living in the community, while, for residents, the main determinants of quality of life are chronic diseases.	Q2 61.5%

[35]	E. Transversal Retrospective	Comparison of home‐based and research‐center assessments of physical performance and activity.	Older adult population with cohorts: 75, 80, and 85 years. *n* = 496	Finland—home and research center	YALE scale. Accelerometry	AF. Gait speed. Gripping strength.	Compare correlations between a variety of measures of physical performance and activity that assess the same underlying construct in different settings	Older people with poorer health and functioning likely refrained from participating in later phases of the study, each of which required more effort or commitment from participants. Paired measures of walking speed (*R* = 0.69), hand grip strength (*R* = 0.85), time of physical activity of at least moderate intensity (*R* = 0.42), and time in an upright posture (*R* = 0.30) assessed in different settings were correlated with each other, and correlated with indicators of health, functioning, and general activity. Associations were strong regardless of limitations in health and functioning, and low overall activity.	Correlational analyses did not clearly reveal a superior environment for assessing performance or physical activity.	Q1 82%

[13]	Systematic review	Systematic review of movement‐behavior questionnaires and their measurement properties.	Elderly population > 65. *n* = 55 items	Several countries—not applicable (systematic review)	QPPA. GPPAQ. LASA. YALE. PASE. IPAQ.	Dream. Sedentary behavior. AF	To systematically review the literature on the content and measurement properties of self‐reported and indirect reporting questionnaires that measure movement behaviors, or their combination, in adults and older adults.	Data extraction, results, study quality, and risk of bias were assessed using the guidelines of the Consensus‐Based Standards for the Selection of Health Measurement Instruments (COSMIN). Fifty‐five articles were included in this review, describing 60 questionnaires. None of the questionnaires showed adequate criterion validity and adequate reliability, simultaneously; A total of 68.3% showed adequate content validity. The risk of bias for criterion validity and reliability was very low in 72.2% and 23.6% of studies, respectively.	Existing questionnaires have insufficient measurement properties and frequent methodological limitations, and none were developed considering the 24‐h movement behavior paradigm. The lack of valid and reliable questionnaires that assess 24‐h movement behaviors in an integrated manner,	Q1 84.6%

[64]	E.Reliability	Reliability and validity study of the IPAQ against accelerometer cut‐off points.	Elderly population between 60 and 89 years old. *n* = 89 individuals	United Kingdom—community‐dwelling older adults	International Physical Activity Questionnaire (IPAQ ‐ Long). Accelerometry	Moderate‐vigorous PA. Sedentary behavior. Differences between the sexes.	To determine the reliability and validity of sedentary behavior (SB) as measured by IPAQ and moderate‐vigorous physical activity (MVPA) in older people, while examining any sex differences in reliability and validity results.	IPAQ showed weak reliability qualities for total SB (h·week −1) and MVPA 10 min (cumulative in sets ≥ 10 min continuously, h·week −1). IPAQ had poor concurrent validity qualities for total SB, 10‐min MVPA, but not for sporadic MVPA (cumulative in bouts < 10 min continuous, h·week −1). IPAQ only correctly classified participants’ physical behavior 2% of the time. Sex differences were only present for the correlation slope of the reliability measures of the 10‐min MVPA of the IPAQ.	Long IPAQ. Originally validated for people between 15 and 69 years old. Low reliability in older adults (although many studies use it indiscriminately). He is not precise enough in his questions for this population.	Q1 66.6%

[9]	Systematic review	Updated systematic review of measurement properties of self‐administered PA questionnaires.	Adults over 55 years of age. *n* = 56 items	Several countries—not applicable (systematic review)	PASE. PASBQ. ACTIVE Q. QAPPA.	PA	To summarize, evaluate, and compare the measurement properties of all available self‐administered questionnaires assessing PA in older adults	In total, we included 56 articles on 40 different questionnaires (14 from the previous review and 26 from the update). Reliability was assessed in 22 questionnaires, measurement error in four, and hypothesis testing for construct validity in 38 different questionnaires. Evidence of responsiveness was available for one questionnaire. For many questionnaires, only one measurement property was assessed in a single study. Sufficient content validity was considered for 22 questionnaires. All questionnaires showed large measurement errors. Only the two‐questionnaire versions showed sufficient reliability and hypothesis testing for construct validity, namely the Physical Activity Scale for Older People (PASE) for the assessment of total PA and the Physical Activity and Sedentary Behavior Questionnaire (PASB‐Q) for the assessment of MVPA. The quality of the evidence for these outcomes ranged from very low to high.	Talk about all the quizzes in other articles. But two scales showed sufficient reliability and hypothesis testing for construct validity. PASE (to measure total PA) and SBPA (moderate vigorous PA).	Q1 76.9%

[36]	Methodological	French translation, cultural adaptation, and validation of PASE.	Elderly adult population > 65 years of age. *n* = 89 individuals	France—community‐dwelling older adults	Adapted PASE, IPAQ‐SF, SF‐12	PA	To translate the Physical Activity Scale for the Elderly (PASE) questionnaire into French, adapt it to the French European culture and validate it.	The translation faced no major problems, with moderate cultural adjustments. Unfamiliar activities such as American football, shuffleboard or aerobic dancing were adjusted, while common sports like yoga, aqua cycling and electric cycling were added. Validation study involved 89 older participants (median age of 73 (69.5–77) years, 58% of women). Moderate internal consistency was found (Cronbach’s alpha = 0.571). Test‐retest reliability was very good for household activities (ICC = 0.712 (95% CI = 0.496–0.845)) and work‐related activities (ICC = 0.955 (95% CI = 0.908–0.978)) but was lower in the leisure section (ICC = 0.163 (95% CI = − 0.183–0.473)), leading to a moderate overall score (ICC = 0.455 (95% CI = 0.125–0.608)). This result could be attributed to the weather conditions that were not similar between the two test intervals, which affects leisure activities (most of which take place outdoors). Construct validity was almost confirmed (66.67% of the ideal 75% hypothesis was validated). No floor or ceiling effects were detected.	The French PASE appears to be a reliable and relatively valid tool for assessing household and work‐related activities. However, PASE should be used with caution, especially when assessing leisure time activities, ensuring that the meteorological conditions are consistent between the two reliability tests.	Q1 92.3%

[12]	R. Systematics + Meta‐analysis	Systematic review and meta‐analysis of validity and reliability of international PA questionnaires.	General adult population. *n* = 20 items	European Union—not applicable (systematic review/meta‐analysis)	IPAQ. GPAQ. EHIS‐PAQ.	Transnational PA and surveillance systems	To critically assess, compare and summarize the measurement properties (reliability, criterion validity, construct validity) of the most commonly used PAQs in transnational surveillance systems for adults in the official EU language. Versions, taking into account the methodological quality of these studies, as well as the quality of the evidence.	The results indicate that only 10 EU countries validated the official language versions of the selected PAQs. A meta‐analysis revealed that the assessment of moderate‐to‐vigorous PA (MVPA) is the most relevant outcome of the PA level, as no publication bias was detected in any of the measurement properties, while test–retest reliability was moderately high (rw = 0.74), moderate for criterion (rw = 0.41), and moderately high for concurrent validity (rw = 0.72). Information on study methods and outcomes was poor, with an overall moderate risk of bias with a total score of 0.43.	In conclusion, when only self‐report of PA is feasible, assessment of MVPA with selected PAQs in adult populations in the EU is recommended.	Q2 89.8%

[54]	E. Transversal	No intervention; cross‐sectional assessment of dietary intake frequency and physical activity.	Elderly adult population > 85 years of age. *n* = 810 individuals	Croatia—community‐dwelling adults aged 85+	International Physical Activity Questionnaire (IPAQ).	OF. Índice de Dieta	To explore the association between various frequencies of dietary intake and physical activity in older adults	“Optimal” intake of fish and seafood (OR (odds ratio) = 1.40; 95% CI (95% confidence interval) 1.01 to 2.00), fruits (OR = 2.10; 95% CI 1.45 to 3.02), legumes (OR = 1.73; 95% CI 1.19 to 2.50), olive oil (OR = 1.83; 95% CI 1.09 to 3.08) and bread (OR = 4.62; 95% CI 3.05 to 6.99) and the total Diet Index for Older People score (OR = 4.99; 95% CI 3.20 to 7.70) were associated with ‘sufficient’ physical activity. When all components of the diet were simultaneously entered into the model, the “optimal” meat intake (OR = 1.73; 95% CI 1.10 to 2.71), fish and shellfish (OR = 2.26; 95% CI 1.46 to 3.51), cereals (OR = 1.75; 95% CI 1.02 to 3.25), fruits (OR = 1.52; 95% CI 1.02 to 2.26), legumes (OR = 1.48; 95% CI 1.10 to 1.93) and bread (OR = 5.14; 95% CI 3.24 to 3.25). 8.15) were associated with “sufficient” physical activity.	The Senior Diet Index total score is the strongest predictor associated with “sufficient” physical activity in an older adult population.	Q1 84.6%

[52]	Reliability Study	Development and validation of the Activity Diversity Questionnaire	Elderly adult population > 65 years old. *n* = 30 individuals	Japan—community‐dwelling older adults	Diversity of Activities Questionnaire (ADQ).	Diversity of activities (sport, listening to music, instrumental tasks…)	Develop a tool for assessing the diversity of activities using a sufficient variety of activities and verify the validity and reliability of the tool.	Study 1. In total, there were 30 participants (all female), mean age 77 (range 69–92) years, mean years of education (standard deviation; SD) was 11.7 (1.9) years; none had undergone major changes in daily life during the examination period. Table 1 shows diversity scores and CCIs for the first and second administration of the ADQ. The diversity scores of the first and second administrations were 0.73 (range 0.23–0.86) and 0.76 (range 0.43–0.90), the ICC of the diversity scores was excellent (0.84, *p* < 0.01). Study 2. There were 766 participants in total (60.4 women), the mean age was 73.5 (range 65–91) years. The ADQ indicated that the total number of activities carried out during the past week was 10.5 (2.5; range 3–18); the total frequency score was 22.9 (5.5; range 5–40); The diversity score was 0.74 (0.09; range 0.32–0.93). There are correlations between the diversity scores derived from the ADQ and those taken from the Tokyo Metropolitan Institute of Gerontology Competency Index (TMIG‐IC) and Japan Science and Technology Agency Proficiency Index (JST‐IC) as external standards, the correlation coefficients were 0.48 (*p* < 0.01) and 0.60 (*p* < 0.01), respectively.	The ADQ was developed using scientific procedures and revealed sufficient reliability and validity. As such, it is a scientifically validated tool for assessing the diversity of activities among older adults.	Q2 84.6%

[24]	Mixed‐methods analysis of an RCT	Mixed‐methods evaluation of PA measures within an RCT in mild cognitive impairment or mild dementia	Elderly population between 66 and 92 years old. *n* = 49 individuals	United Kingdom—community sample with mild cognitive impairment or mild dementia	International Physical Activity Questionnaire (IPAQ). Amsterdam PA Questionnaire from the Longitudinal Ageing Study (LASA)	AF. Mild cognitive impairment. Motivation.	This study explored the following three questions: (a) what were the completion or return rates for each of the measurements? (b) Is there a relationship between physical activity questionnaire scores, exercise levels reported on the calendar, and physical activity levels recorded on accelerometers? and (c) what was the participants’ experience of using the accelerometers and completing the activity schedule?	Quantitative analysis showed equal completion rates for the International Physical Activity Questionnaire and accelerometer, but a lower completion rate for the calendar. Correlations between outcome measures were moderate or strong. Qualitative analysis indicated that all measures were acceptable, although some participants required assistance in completing schedules or holding accelerometers. The study supported the validity of these methods for people with mild cognitive impairment and mild dementia.	Using accelerometers and completing activity calendars can increase motivation to be active for some people.	Q3 61.5%

[58]	Methodological	Development and psychometric validation of the Perceived Physical Literacy Questionnaire	Elderly population between 60 and 88 years old. *n* = 388 individuals	China—community‐dwelling older adults	PPLCEQ questionnaire	Physical activity. Physical literacy. Validation. Reliability.	To develop the Perceived Physical Literacy for Chinese Elderly Questionnaire (PPLCEQ) and evaluate its psychometric properties.	The developed PPLCEQ includes 47 items. Consistent with the conceptual definition of physical literacy, exploratory factor analysis showed that the PPLCEQ is composed of 6 subscales. Participants’ PPLCEQ scores were moderately correlated to their leisure‐time PA (*r* = 0.38, *p* < 0.001), PA maintenance (*r* = 0.44, ps < 0.001), and perceived competence for exercising regularly scores (*r* = 0.58, *p* < 0.001). Moreover, the Cronbach’s alpha and the test–retest reliability of the questionnaire were 0.88 and 0.70, respectively.	Psychometric assessment results suggest that the PPLCEQ is a reliable and valid tool that can be used in future studies investigating Chinese older adults’ perceived physical literacy.	Q1 84.6%

[57]	Mixed Methodological	Development and validation of a physical activity barriers assessment scale	Elderly adult population > 65 years old. *n* = 523 individuals	China—hospitalized older adults with chronic diseases	Physical activity barriers scale; Interviews	Physical activity. Barriers. Validation.	To develop and validate a Physical Activity Barrier Assessment Scale for this population.	The final 29‐item scale comprises three domains: individual (health status, psychological factors), interpersonal (caregiver/staff support), and organizational (environmental barriers). EFA revealed four components explaining 74.14% variance. CFA indicated an acceptable model fit (RMSEA = 0.062; CFI = 0.834). The scale demonstrated excellent internal consistency (Cronbach’s *α* = 0.863). Content validity index reached 0.931.	The scale demonstrates robust psychometric properties for identifying physical activity barriers in hospitalized older adults with chronic diseases. This instrument enables healthcare providers to develop targeted activity interventions, potentially improving clinical outcomes in this vulnerable population.	Q1 100%

[34]	E. Transversal	Cultural adaptation and validation of the Polish PASE.	Elderly adult population > 65 years of age. *n* = 115 individuals	Poland—community‐dwelling older adults	Polish Physical Activity Scale for the Elderly (PASE‐P). International Physical Activity Questionnaire (IPAQ). Accelerometers.	AF. Level of independence. Instrumental activity. Balance.	Provide cultural adaptation and validation of the Polish version of the Physical Activity Scale for Older People (PASE‐P).	The mean P‐PASS was 91.54 (SD 71.15). Among the trials, we found sufficient reliability of the test–retest of the components of the PASE‐P questionnaire. The ICC test was robust and ranged from 0.988 to 0.778 for both the main domains and the total scale score. A significant correlation was found between the total PASE‐P score and the shortest TUG, the TUG gear (*r* = − 0.514, *p* < 0.001; *r* = − 0.481, *p* < 0.001) and 10MWT (*r* = 0.472, *p* < 0.001). The total PASE‐P score was also positively correlated with ADL and IADL (*r* = 0.337, *p* < 0.001; *r* = 0.415 *p* < 0.001), BBS (*r* = 0.537, *p* < 0.001), and 5xSTS (*r* = 0.558, *p* < 0.001).	PASS‐P. It was short, easy to qualify, understandable, and relevant to the culture of Polish older adults. It demonstrated good test–retest reliability and the quality of a valid tool capable of assessing the level of PA among older adults living in communities in Poland. High correlation with IPAQ, Katz Index, and low correlation with TUG.	Q1 61.5%

[28]	E. Transversal	No intervention; online survey of changes in physical activity during COVID‐19.	Elderly adult population > 65 years of age. *n* = 1600 individuals	Japan—community‐dwelling older adults (online survey)	International Physical Activity Questionnaire (IPAQ ‐ Short). Kihon Frailty Checklist	BY. Nivel Fragilidad. Pandemia	To investigate changes in physical activity (PA) between January (before the COVID‐19 epidemic) and April (during the COVID‐19 epidemic) 2020 in older adults living in communities in Japan.	The mean age of the study participants, the proportion of women, and the prevalence of frailty were 74.0 ± 5.6 years, 50% (*n* = 800), and 24.3% (*n* = 388), respectively. We found a significant decrease in total time spent physical activity in April 2020 (median [interquartile range (IQR)], 180 [0 to 420]) compared to January 2020 (median [IQR], 245 [90 to 480]) (*p* < 0.001). We also performed a subgroup analysis according to the category of frailty; Total time spent physical activity decreased significantly in April 2020 compared to January 2020 for all frailty categories (*p* < 0.001).	Due to the COVID‐19 epidemic, total physical activity time in April 2020 decreased significantly compared to January 2020 in older adults. This finding may lead to a higher incidence of disability in the near future in older people	Q1 82%

[27]	E. Transversal	No intervention; follow‐up online survey of pandemic‐related physical activity decline and incident frailty.	Elderly adult population > 65 years of age. *n* = 937 individuals	Japan—community‐dwelling older adults (online follow‐up survey)	International Physical Activity Questionnaire (IPAQ ‐ Short). Kihon Frailty Checklist (KCL).	BY. Nivel Fragilidad. Pandemia	To investigate the influence of the COVID‐19 pandemic on physical activity (PA) and the incidence of frailty among initially nonfrail older adults in Japan.	Total physical activity time during the first, second, and third waves of the pandemic decreased from prepandemic physical activity time by 33.3%, 28.3%, and 40.0%, respectively. Notably, the total physical activity time of older adults living alone and socially inactive decreased significantly: 42.9% (first wave), 50.0% (second wave), and 61.9% (third wave) less than before the pandemic, respectively. In addition, they had a significantly higher risk of frailty incidents than those who did not live alone and were socially active (adjusted odds ratio: 2.04 [95% confidence interval: 1.01–4.10]).	Older adults who live alone and are socially inactive are more likely to experience incidents of frailty/disability due to decreased physical activity during the pandemic	Q1 82%

### 3.3. Co‐Occurrence Analysis

**FIGURE 4:**
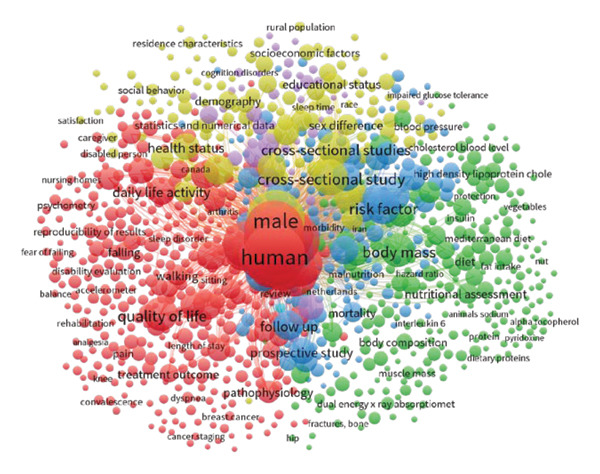
Co‐occurrence analysis.

In addition, co‐occurrence analysis showed how some concepts related to PA in the older adult emerged in the network, such as male, human, risk factors, activity diary, body mass, diet, monitoring, and research studies. In addition to the term cross‐sectional, the prevailing items in the network were knowledge and research. Finally, quality of life and health status were components related to PA (Figure 4).

## 4. Discussion

The relationship between PA and health in older adults constitutes a complex interplay where movement patterns and sedentary behavior directly dictate quality of life. This review confirms that assessing PA in this population requires a transition from narrative description to a standardized, multidimensional approach that considers the interaction between PA, sleep, nutrition, and social participation to develop effective public health strategies. As noted by Miezah et al. [59], the choice of assessment instruments must align with the specific psychometric strengths and the population’s characteristics, moving beyond a “one size fits all” model.

### 4.1. Integration of Assessment Tools, Measurement Challenges: From Questionnaires to 24‐h Monitoring

A significant portion of current research remains focused on the validation of self‐report tools. Although the IPAQ, CHAMPS, and PASE continue to be the most widely used [65], recent evidence from authors such as Ayvat et al. [31] and Doğan et al. [30] warns that these scales have a limited ability to discriminate frailty states and should not be used interchangeably, as they capture distinct aspects of behavior.

Furthermore, the adaptation of tools for specific cultural or linguistic contexts, such as the Persian RAPA [37], the Polish PASE‐P [34], Japon PASE [66], or the French PASE [36], highlights the need for precision. The discrepancies between self‐reports and accelerometry emphasized by Domingos et al. [41]and Rodrigues et al. [13] reinforce the necessity of 24‐h movement monitoring through electronic devices to eliminate self‐assessment bias.

To overcome self‐assessment biases, there is a pressing need to monitor movement over a 24‐h period using electronic devices [13]. In this regard, digital innovation through Ecological Momentary Assessment (EMA) triggered by sensors represents the current frontier for capturing real‐time data. This allows for a deeper understanding of not only how much an older adult moves but also the specific context in which that movement occurs [46].

### 4.2. PA as a Determinant of Clinical and Functional Outcomes: Pain, Fatigue, and Frailty

The evidence gathered consistently shows that PA serves as a protective factor against chronicity. Geneen et al. [67]and Hirase et al. [25] link PA to improved quality of life in patients with chronic pain and frailty. In terms of functional performance, Portegijs et al. [35] and Özler et al. [68] demonstrate that walking speed and moderate PA are essential for postural stability and fall prevention.

Notably, there is an inverse relationship between functional performance and fatigue; the use of specific tools such as the *Pittsburgh Fatigability Scale* (PFS) has confirmed that moderate PA significantly reduces exhaustion and increases vitality [33, 69]. Furthermore, nonexercise PA (ADL) demonstrates a preventive role against frailty similar to structured exercise, which is vital for patients with severe functional limitations [32].

The impact of PA extends to cognitive and emotional health. Gu et al. [22] and Van der Wardt et al. [24] provide evidence of a dose–response relationship between PA and brain volume, even in populations with mild cognitive impairment. Moreover, the role of PA in mitigating fatigue [33, 69] and frailty [16, 32] underscores its systemic benefit.

### 4.3. Biopsychosocial Factors: Nutrition, Social Participation, and Environment

This review identifies that the impact of PA varies drastically depending on the living environment. In the community, nutritional status and PA are the pillars of quality of life; however, in institutional settings, chronic diseases predominate as health determinants [29]. PA does not act in isolation. Papadopoulou et al. [53] and Pigłowska et al. [29] emphasize a holistic approach where nutritional status and sleep quality are critical predictors of health. Social factors also play a decisive role; loneliness is linked to decreased PA [70], while higher socioeconomic status and social participation promote engagement in leisure activities, reducing frailty risk [10, 55].

The distinction between community‐dwelling and institutionalized aging is also vital. Akosile et al. [14] suggest that aging at home offers better health outcomes and less fear of falling, which should guide future public health strategies toward aging‐in‐place models.

### 4.4. Efficacy of Multicomponent Interventions

Regarding the intervention‐related findings, the results show a predominance of observational studies and questionnaire‐based assessments [14, 49, 60], along with studies focused on instrument validation [1, 37, 47], indicating a limited presence of structured interventions. This pattern reflects a research trend in which the assessment of PA continues to be prioritized over the development of intervention programs in older adults.

However, some included studies do implement interventions, such as home‐based exercise programs [39] or those examining behavioral changes in PA [16, 32], highlighting the potential of these strategies. These findings are consistent with recent evidence emphasizing the effectiveness of multicomponent exercise in improving functional capacity and quality of life in older adults [70].

Furthermore, findings linking PA with health‐related variables such as frailty, quality of life, and overall health status [10, 26, 53] align with recent research highlighting its role as a key determinant in reducing mortality risk and maintaining health in aging populations [71].

Overall, these findings highlight the need to strengthen the development of intervention‐based studies, as, although the benefits of PA are well established, their implementation through structured programs remains limited within the analyzed literature.

### 4.5. The Evidence Paradox: Assessment vs. Intervention

A critical finding of this scoping review is the disproportion between studies dedicated to instrument validation and the actual implementation of intervention programs. The literature reflects a tendency to prioritize “how to measure” over “how to intervene.”

Nonetheless, the interventions that have shown success are multicomponent in nature (strength, balance, and educational support). Programs such as home‐based exercises [39] and those integrating perceived physical literacy [58] demonstrate that improving autonomy is achievable. The efficacy of these strategies lies in their ability to adapt to the participant’s functional capacity and be sustained over time, transforming sedentary behavior into a preventive opportunity [16].

### 4.6. Proposed Conceptual Framework for Clinical and Community Implementation

Based on the synthesis of this evidence, we propose a multidimensional framework to guide future research and clinical practice:

• Comprehensive assessment: Clinical practice should move toward a “24‐h behavior” model, integrating objective sensors (where feasible) with validated questionnaires like the GPAQ or PASE, depending on the objective [9, 12]. It is not enough to measure PA; it is imperative to evaluate perceived barriers (pain, fatigue, and fear) using robust scales such as the *Physical Activity Barrier Assessment Scale* [57].
• Targeted interventions: Programs must address barriers to PA [57] and include balance, dynamic stability, and nutritional education to maximize quality of life [26, 27].
• Digital and ecological innovation: The use of sensor‐triggered ecological momentary assessment [46] represents the future of real‐time data collection, allowing for more precise and personalized interventions.
• Policy, technology, and social strategy: Public health policies should foster inclusive environments and accessible leisure activities using tools like the LVAT‐E [51] or ADQ [52], to activate social participation as a pillar of healthy aging.
• Public health policies: It promotes safe and accessible environments that guarantee equitable access to active aging programs, integrating PA into standard clinical pathways.

In conclusion, this review advocates for the integration of evidence‐based components into a biopsychosocial model of aging. Strengthening the connection between accurate measurement tools and individualized interventions is essential for promoting autonomy and well‐being in the global older adult population.

### 4.7. Limitations

This scoping review presents several limitations that should be considered when interpreting the findings. First, the heterogeneity of the included studies in terms of design, population characteristics, assessment tools, and contexts (community, clinical, and residential settings) limited the possibility of direct comparison and synthesis of results. Second, a large proportion of the included studies were observational or focused on the validation of assessment instruments, while relatively few implemented structured intervention protocols, which restricts the ability to draw strong conclusions about intervention effectiveness.

Additionally, the reliance on self‐reported PA questionnaires in many studies may introduce recall and reporting bias, despite their practicality in large populations. Although some studies incorporated objective measures such as accelerometry, these were not consistently applied across all studies. Another limitation is the restriction to studies published between 2019 and 2026 and in English or Spanish, which may have excluded relevant evidence from other periods or languages.

Finally, as a scoping review, this study aimed to map the existing evidence rather than to assess the effectiveness of interventions or establish causal relationships. Therefore, the findings should be interpreted as an overview of the current landscape rather than definitive evidence for clinical decision‐making.

## 5. Conclusion

This scoping review highlights the complexity of PA in older adults, emphasizing the need for a multidimensional approach that integrates assessment tools, intervention strategies, and biopsychosocial factors associated with PA. The findings show a wide variety of validated instruments, with the **Physical Activity Scale for the Elderly (PASE)**, the **International Physical Activity Questionnaire (IPAQ)**, and the **CHAMPS questionnaire** emerging as the most frequently used tools across different contexts.

Although these instruments demonstrate acceptable reliability and feasibility, their applicability varies depending on population characteristics and settings, reinforcing the need for careful and context‐specific selection. Despite the well‐established benefits of PA on functional capacity, quality of life, and health outcomes, the limited number of structured interventions identified suggests a gap between evidence and its practical implementation.

Multicomponent and tailored interventions, particularly those combining physical, behavioral, and social elements, appear to be the most promising approach. Future research should prioritize the development of intervention‐based studies, the integration of objective and subjective assessment methods, and the application of a biopsychosocial framework to better understand and promote PA in older adults. Strengthening these aspects will be essential to support healthy aging and improve autonomy and well‐being in this growing population.

## Funding

This study was funded by the IBIMA Plataforma Bionand, Instituto de Investigación Biomédica de Málaga.

## Conflicts of Interest

The authors declare no conflicts of interest.

## Supporting Information

Additional supporting information can be found online in the Supporting Information section.

## Supporting information


**Supporting Information** The supporting and complementary materials associated with this manuscript provide methodological and structural transparency for this scoping review. Below is a brief description: This document contains the Preferred Reporting Items for Systematic Reviews and Meta‐Analyses extension for Scoping Reviews (PRISMA‐ScR) checklist. It provides a detailed, item‐by‐item verification mapping (Items 1 to 22) corresponding to the methodological reporting transparency, source selection, data charting, and synthesis methods utilized in this review.

## Data Availability

The data that support the findings of this study are available from the corresponding author upon reasonable request.
